# Cross-Sectional Study of Plant Sterols Intake as a Basis for Designing Appropriate Plant Sterol-Enriched Food in Indonesia

**DOI:** 10.3390/nu13020452

**Published:** 2021-01-29

**Authors:** Drajat Martianto, Atikah Bararah, Nuri Andarwulan, Dominika Średnicka-Tober

**Affiliations:** 1 Southeast Asian Food and Agricultural Science and Technology (SEAFAST) Center, IPB Darmaga Campus, IPB University, Bogor 16680, West Java, Indonesia; drajat_martianto@yahoo.com (D.M.); andarwulan@yahoo.com (N.A.); 2 Department of Community Nutrition, Faculty of Human Ecology, IPB Darmaga Campus, IPB University, Bogor 16680, West Java, Indonesia; 3 Department of Food Science and Technology, Faculty of Agricultural Technology, IPB Darmaga Campus, IPB University, P.O. Box 220, Bogor 16680, West Java, Indonesia; atikah.bararah@gmail.com; 4 Department of Functional and Organic Food, Institute of Human Nutrition Sciences, Warsaw University of Life Sciences, Nowoursynowska 159c, 02-776 Warsaw, Poland

**Keywords:** phytosterols, fortification, functional food, cardiovascular risk, cholesterol

## Abstract

Coronary heart disease (CHD) is one of the leading causes of mortality in many low-income and middle-income countries, including Indonesia, with elevated blood cholesterol level being one of significant risk factors for this condition. The problem should be addressed by combining healthy lifestyle and diet, where functional foods having a cholesterol-lowering activity could play a significant role. A group of compounds that had been proven to show cholesterol-lowering ability are plant sterols. To develop more suitable functional foods that could substantially contribute to hypercholesterolemia prevention in Indonesian population, up-to-date data about plant sterols dietary intake are required, and were not available until this research was done. This study aimed to estimate daily plant sterols intake and to determine the consumption pattern of foods containing plant sterols in rural and urban area of Bogor, West Java, Indonesia. The research was conducted with a cross-sectional design, with 200 respondents. The study revealed that the level of plant sterols intake in Bogor reached on average 229.76 mg/day and was not significantly different between urban and rural area. Cereals, vegetables, and fruit products were the main food sources of plant sterols in both areas. In addition, a list of several surveyed food items possible to be enriched with plant sterols was developed within the study. These results provide baseline data to develop functional foods fortified with plant sterols suitable for the Indonesian needs and taste. However, further studies are needed to confirm efficacy and safety of introducing such phytosterol-enriched products into a habitual diet, especially considering possible long-term side effects of plant sterol treatment.

## 1. Introduction

The burden of non-communicable diseases (NCDs) has recently become a major public health concern globally, with NCD-related mortality figures rising dramatically in low-income and middle-income countries [1,2,3,4]. In Indonesia, cardiovascular diseases are currently estimated to cause more than 30% of all deaths, with coronary heart disease (CHD) and stroke diagnosed as the leading causes of deaths in the country [4,5,6,7,8]. In 2018, Indonesia was the country with the second highest number of disability-adjusted life years (DALYs) lost to CHD [9,10].

There are many risk factors for CHD, including elevated blood cholesterol level, high blood pressure, smoking, overweight and obesity, diabetes, unhealthy diet, lack of physical activity, and stress [11,12]. Consistent evidence from numerous clinical and genetic studies unequivocally establishes that LDL cholesterol is one of the important causal factors in the pathophysiology of atherosclerotic cardiovascular disease (ASCVD), leading to, i.e., CHD and strokes [13]. Thus, lowering LDL cholesterol level is considered as one of efficient and effective strategies to reduce the risk of CHD and other hypercholesterolemia-related diseases [13,14,15,16,17].

Plant sterols and stanols, also known as phytosterols, form a group of compounds with well-proven cholesterol-lowering ability [18,19,20,21]. Natural plant sterols are found in vegetables and vegetable-based foods, including vegetable oils and vegetable oil-based margarines, but also in nuts, seeds, cereal products, legumes, and some fruits, most of which are widely consumed by Indonesian population. Despite the availability of healthy plant-based foods, the trend of fast food is now becoming an alarming condition. As a counterweight, functional foods with cholesterol-lowering ability could be considered relevant to be consumed side-by-side with daily food, in order to contribute to the prevention of CHD and other hypercholesterolemia-related diseases.

Several proposed mechanisms of plant sterols action in lowering blood LDL cholesterol level have already been scientifically proven [15,16,18,22]. A clinically significant LDL cholesterol-lowering effect of about 10% can be achieved by a daily intake of 1.5 to 3 g of plant sterols [18]. The American Heart Association Nutrition Committee also released similar recommendation of plant sterols intake equal to ~2 g/day, with consumption of more than 3 g/day indicated as having no further beneficial effect in blood cholesterol level [23]. Following these recommendations, daily consumption of plant sterol rich foods has been indicated as a potentially effective strategy contributing to LDL cholesterol reductions.

However, it should be pointed that while many renowned international associations such as the European Atherosclerosis Society (EAS) recommend the use of plant sterols and stanols as food additives to reduce blood cholesterol levels [24], the new guidelines of such organizations as, e.g., the European Cardiology Society, the German Drug Commission, and the National Institute of Health and Clinical Excellence (NICE) in the United Kingdom underline that long-term studies are still needed to guarantee the safety of phytosterol-enriched products [25,26], especially considering possible long-term side effects of plant sterol treatment (i.e., their potential contribution to atherosclerosis) [27].

Although several food products enriched with plant sterols are already available in the market, more affordable, safe, and effective products for hypercholesterolemia prevention are still highly required. To facilitate the potential development of functional foods to be consumed side-by-side with daily meals by common Indonesian population, supporting scientific data should be available. Until this research was done, there was no study on the consumption pattern of food containing plant sterols by general population in Indonesia. Thus, the intake of plant sterols and the contribution of dietary plant sterols to daily consumption have never been measured. The reported study provides the portrait of consumption patterns of foods containing plant sterols in Indonesia, as well as the information on plant sterols intake in Indonesian population, as a baseline for potential development of appropriate functional food products enriched with plant sterols that could be afforded, accepted, and that would meet Indonesian taste.

## 2. Materials and Methods

### 2.1. Study Design

The study was conducted with a cross-sectional design similar to several other studies [28,29,30]. It was located in Bogor (West Java, Indonesia), consisting of urban and rural areas. Rural and urban areas of Bogor were selected due to the 12.1% and 7.8% higher prevalence of CHD compared with other districts in West Java Province, respectively [31]. 

The target population comprised of 200 adult respondents, including 100 persons in each area (urban and rural): 50 men and 50 women. Respondents were selected based on several inclusion criteria verified with the use of a questionnaire: willingness to participate in the study, age between 25 and 65 years, body mass index (BMI) between 18 and 27, blood pressure between 90/60 and 140/90 (systole/diastole), and not being in the treatment of any disease. A detailed respondent selection flowchart has been presented in Figure S1 (Supplementary Materials). The study was conducted according to the guidelines of the Declaration of Helsinki, and was approved by the Institutional Review Board of School of Medicine, Diponegoro University, Indonesia (protocol code No. 247lEC/FK/RSDK/2012, date of approval 18 July 2012).

### 2.2. Respondents’ Characteristics and Dietary Data (Plant Sterols and Nutrients Intake)

Basic information about respondents, including socio-demographic and individual characteristics, as well as dietary data, were obtained through interviews conducted by trained staff, with the use of 2-day semiquantitative food recall (2 × 24 h from the day of interview backwards) and food frequency questionnaire (FFQ). Respondents also completed a list of several plant sterols containing food products in FFQ. Basic respondents characteristics are presented in Table S1 (Supplementary Materials).

### 2.3. Plant Sterols Content in Foods

The amount of each food item consumed was converted to grams and its plant sterol content was calculated. As done by Andersson et al. [28] and Klingberg [32], the calculations were based on USDA database and data available in scientific publications. However, for some foods consumed by respondents for which data was not yet available, several methods were applied: (a) values assigned from similar products listed in secondary data, (b) values assigned based on calculations of standard recipes with ingredients listed in secondary data (with the recipes obtained from interview), and (c) plant sterols content set to zero due to pure animal origin of the product or ingredients not containing plant sterols.

### 2.4. Blood Cholesterol Level

Total blood cholesterol level of respondents was tested through a fingertip test using standard cholesterol home test kit (The CardioChek PA analyzer, PTS Diagnostics ©, Indianapolis, IN, USA).

### 2.5. Data Processing and Analysis

Data on food consumption obtained by 2-day (2 × 24 h) food recall were converted to grams, and then food and nutrients daily intake was calculated using the Indonesian Food Composition Data (DKPI) [33]. Based on the information obtained from the Food Frequency Questionnaires, food products were categorized into 12 groups: (1) beverages, (2) cereals and cereal products, (3) eggs and egg products, (4) fish and fish products, (5) fruits and fruit products, (6) herbs, spices and condiments, (7) meat and meat products, (8) legumes, legume products and nuts, (9) plant sterols fortified products, (10) snack foods, (11) supplements, and (12) vegetables and vegetable products.

The differences in food and plant sterols consumption between (a) rural and urban areas and (b) male and female participants were analyzed statistically using the independent samples *t* test. Additionally, Pearson’s product–moment correlation analysis was carried out to identify potential linear associations between the calculated plant sterols intakes and total blood cholesterol levels in the studied population. All statistical analyses were performed using SPSS PASW v. 18 software (SPSS Inc., Chicago, IL, USA).

### 2.6. Supplements and Functional Foods Enriched with Plant Sterols Available on the Market

All plant sterol supplements and functional foods enriched with plant sterols, or acclaimed to be fortified with plant sterols, whether intended to hypercholesterolemia or CHD patients or not, were surveyed. Their availability in Indonesian market was surveyed with two steps: websites screening and direct survey of the market around the study area.

## 3. Results

### 3.1. Food and Nutrients Daily Intakes

Total daily food consumption in the rural area of Bogor was significantly higher compared to the urban area. Both in the rural and urban areas, food consumption in the group of male respondents was higher than in the group of female respondents. Intake of energy, protein, iron, vitamin A, and vitamin C was also analyzed. Despite the smaller amount of food consumed daily by urban compared to the rural respondents, the levels of the analyzed nutrients intake in both areas were comparable. There were several gender-related differences in particular nutrients intake. In rural area the intake of both vitamin A and vitamin C was significantly higher in the group of female compared to the male respondents. This trend was not so strong in the urban area for vitamin C, and opposite (with higher intake in the group of male vs. female respondents) for vitamin A (Table 1).

The consumption data for each food group are shown in Table 2. The most consumed food group in both the rural and the urban area was cereals and cereal products, followed by vegetables and vegetable products, fruits and fruit products, and snack foods. Consumption of foods predicted to contain plant sterols in urban area was slightly higher compared to the rural area, although the difference was not statistically significant (*p* > 0.05). Generally, the pattern and amount of food consumption between respondents from both areas was similar.

### 3.2. Plant Sterols Daily Intake

Calculation of the average plant sterols content in each food group required detailed information on the content for each food item from the group. Average plant sterols content in each food group and sub-group consumed by the respondents is presented in Table S2 and in Figure S2 (Supplementary Materials). Composite food products required the respondents to include the recipes. To calculate the plant sterol level in these food items, information on the level of plant sterols in each of the ingredients was needed. As done in previous studies, all the calculations were based on USDA database and plant sterol data available in scientific publications [34]. Detailed information on the plant sterols content in every food item consumed by the respondents, examples of plant sterols content calculations based on product or meal recipe, and all data on the plant sterols content of composite meals based on such calculations are presented in Tables S6–S8 (Supplementary Materials), respectively. The plant sterol content of all products of animal origin was set as zero, as was that of the following products: soft drinks, tea, coffee, cocoa drinks, sugar, honey, syrup, soy sauce, and local herbal drinks [15,32,35]. From the total of 309 food items consumed by respondents, 8.74% of the products had a plant sterol content of zero. There were several food items which probably contain plant sterols, such as papaya, cassava leaves, and other indigenous leaves (Table S3, Supplementary Materials), but yet there were no valid data on their plant sterols content available, thus plant sterols content in these foods was temporarily set to zero. The contribution of particular plant sterol data sources used in the study is presented in Table 3. 

**Figure 1:**
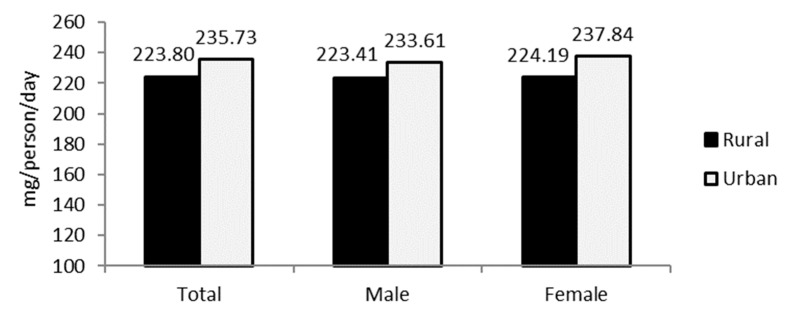
Plant sterols intake in the groups of male and female respondents from rural and urban Bogor area.

The calculated overall plant sterols intake in the studied population reached on average 229.76 mg/day (235.73 mg/day and 223.80 mg/day in the group of urban and rural respondents, respectively). The gender-related differences in plant sterols consumption were not statistically significant (*p* > 0.05) (Figure 1).

In this study, cereals were the main source of plant sterols for all respondents (37.46% and 37.21% in the case of rural and urban respondents, respectively), followed by legumes and legume products (24.41% and 23.59%), and snacks foods (15.33% and 15.42%). The calculated plant sterols intake from each of the food groups by respondents representing rural and urban population of Bogor is depicted in Table 4. The percentage contribution of the plant sterols from each of the consumed food groups to the overall plant sterols intake by rural and urban respondents is additionally shown in Tables S4 and S5, respectively.

### 3.3. Blood Cholesterol Level

**Figure 2:**
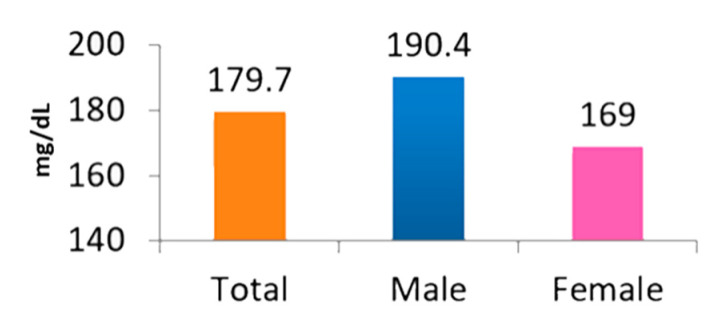
Total blood cholesterol level of the studied population in Bogor area, Indonesia.

The average level of total blood cholesterol in the studied population reached 179.70 mg/dL (190.4 mg/dL in men and 169.0 mg/dL in women) (Figure 2). Pearson’s correlation test did not identify any statistically significant associations between daily consumption of plant sterols and total blood cholesterol level of the study participants (*p* > 0.05).

### 3.4. Plant Sterols Supplements and Fortified Products Available in the Bogor Market

There were several supplements of plant sterols as well as food products fortified with plant sterols or stanols identified in the Bogor market. Those products could serve as a significant source of plant sterols in the diet, although very few respondents declared that they consume these products, mainly due to their limited awareness, unaffordable prices, and inconvenient methods of consumption. In case of the identified products, plant sterols content for each serving size varied from 40 mg to 1.7 g, with a considerable variability in the suggested serving size per day for each product. The list of the identified products is shown in Table 5.

## 4. Discussion

### 4.1. Plant Sterols Daily Intake

This study provided initial data on plant sterols intake in Indonesia, specifically in Bogor rural and urban areas. Bogor rural and urban areas have different administrative boundaries, central governance, and considerably different socio-economic characteristics. However, despite the administrative boundaries, both areas are geographically adjacent, with Bogor urban area located in the middle of the rural area. The contiguous area of both appears to result in similar consumption patterns of their populations, especially near the boundaries. The difference in plant sterol intake between respondents representing both areas was lower than expected and not significant (*p* > 0.05). These findings are similar with the research of Hoffmann et al. [36] about daily food consumption in rural and urban area of the district Szamotuły in Poland, which identified comparable consumption patterns between both areas.

Plant sterol intake was slightly higher in the group of women than in the group of men respondents, both in rural and urban areas. However, this gender-related difference in each area was not statistically significant (*p* > 0.05). In the rural area, most of the men work as farmers and tend to work near residential area, thus they tend to eat food prepared at home. At the same time, although the Indonesian urban residents usually eat out a lot [37], respondents in the Bogor urban area prefer to purchase their meals in small food stalls around their workplace. Such food stalls provide food similar to this prepared at home, with relatively affordable prices. 

Cereals have been shown to be the food group contributing the most to the daily plant sterols intake in many populations. Research in Finland reported that 40% of plant sterols intake comes from cereals [35]. Likewise, 37% of the total plant sterols daily intake was confirmed to be provided from cereals and breads in the Netherlands [15]. A study conducted in China also showed that 40% of plant sterols intake was obtained from cereals and another 40% from vegetable oils [38]. Likewise, the present study also showed that cereals were the main source of plant sterols to all groups of respondents (37.46% and 37.21% in rural and urban area, respectively).

Despite the mentioned resemblance, Indonesia has a relatively low intake of plant sterols compared with other countries. Plant sterol intake in Finland was estimated to reach 305 mg/day for men and 237 mg/day for women [35], while in the UK it was 300 mg/d for men and 293 mg/day for women [32]. In China, adult men and women also had higher plant sterol intake (330 mg/d and 311 mg/d, respectively) [39]. This is probably due to the considerable differences between the diet and types of foods consumed by the Indonesians and by people in other countries, even within the same food group. For examples, cereals consumption in Indonesia is dominated by white rice instead of various types of cereals such as whole wheat, rye, and other cereals with higher plant sterols content, which are widely consumed in other countries [40].

According to the recent estimations, intake of plant sterols from natural sources generally ranges between 200 and 400 mg/day with habitual diets [41] and up to 600 mg with vegetarian and vegan diets [42]. Higher intakes can be achieved by introducing food products enriched with plant sterols into a diet.

### 4.2. Association between Plant Sterols Intake and Blood Cholesterol Level

Based on the Pearson’s correlation test, there was no significant association between the daily consumption of plant sterols and total blood cholesterol level in the studied population. There are several reasons/aspects that could explain this result. Respondents were selected based on several inclusion criteria, such as body mass index (BMI) in a range of 18 to 27, blood pressure between 90/60 and 140/90 (systole/diastole), and not being in the treatment of any disease. Therefore, the results of the study were in fact limited to a healthy population. No association means in fact that there was no dose-dependent effect of the plant sterols consumption on total blood cholesterol (a) in healthy respondents, (b) with the average blood cholesterol level of 179.70 mg/dL, and (c) considering the generally low plant sterols intake (229.76 mg/day) in the studied population. It could be also considered that daily plant sterols intake contributed to keeping blood cholesterol in desirable level in healthy respondents. This is in line with research conducted by Hendriks et al. (1999) [43] who stated that consumption of 200–400 mg of plant sterols per day can maintain the blood cholesterol in desirable level (<200 mg/dL). A study by Berger et al. (2004) [44] showed that free plant sterols found naturally in food can have visible effect in the reduction to LDL cholesterol level by 5% or more at the consumption level of 0.8–1 g/day. In addition, Carr et al. (2010) [45] stated that plant sterols intake from daily food can reduce the absorption of cholesterol, but that doses of 1.5–3 g/day are required to achieve maximal reductions in serum cholesterol.

It should be also underlined that many characteristics of respondents, such as level of education, type of jobs, and income per capita, were similar. This similarity was reflected in their food choices. Thus, they did not have much variation in overall food patterns, including plant sterols intake, and therefore also in the blood cholesterol levels. Moreover, total blood cholesterol level of respondents was tested through a fingertip test using cholesterol home test kit. Such a method can be used for general screening for the hypercholesterolemia risk in the population and may be useful for periodical controlling of blood cholesterol levels [46]. However, to determine the effectiveness of plant sterols intake, a complete plasma lipid profile (including LDL cholesterol, HDL cholesterol, and triglycerides) should be tested, as done by Andersson et al. (2004) [28].

### 4.3. Recommendations of Food Formulation for Maintaining Healthy Blood Lipid Profile

Preventing hypercholesterolemia is one of the important elements of coronary heath disease and other cardiovascular diseases prevention. Various ways are generally proposed for this purpose (including various lifestyle and dietary factors), one of them being a consumption of natural cholesterol-lowering agents such as plant sterols and stanols. Based on the findings summarized by American Heart Association [23], a minimum intake of 1 g of plant sterols per day is already considered to show a cholesterol-lowering effect, and the maximum cholesterol-lowering effect can be achieved by consuming 2–3 g/day. However, the findings of the present study have shown that the intake of plant sterols in the Indonesian population is far below the above-mentioned cholesterol-lowering levels, and thus achievement of such an effect would require a diet modification, including, e.g., the introduction of rich sources of plant sterols, fortified products, and/or supplements.

To address the problem of the increasing prevalence of hypercholesterolemia-related diseases in Indonesia, some plant sterol-enriched food products and supplements have emerged in the Indonesian market, as shown in Table 5 before. Although the products are available in reachable places, most of them are not affordable and less convenient to be taken regularly by common people, especially in rural areas, which in fact are characterized by higher coronary heath disease prevalence and lower plant sterols intake. Due to the lower level of education and lower nutritional and health awareness rural people would be more likely to consume functional food products in the form resembling their usual foods, with the emphasis on price, taste, and ease of consumption [45].

Products enriched with plant sterols that are marketed on the European Union and USA market include i.e., juices, ice creams, snack bars, white, and whole-grain breads and buns, cereals, confectionery products and cooking oils. In addition, GRAS (Generally Recognized as Safe) status was given to plant sterols and plant sterol esters as ingredients in ground roasted coffee, pasta, noodles, soups, puddings, and egg products [47]. In accordance with GRAS status and the possibilities for such enrichment, this study suggests several foods which could be potentially enriched, to provide a considerable cholesterol-lowering effect for the Indonesian population. 

Although several recent findings showed that the efficacy of esterified plant sterols as cholesterol-lowering agents is independent of the food matrix [23,45,48], fat-based foods, such as fat spread or oil, are generally considered to be more preferable in solubilizing plant sterols [48]. In the present study, fat spreads (i.e., margarines) and sauces (i.e., spicy sauce) were minor, yet important, components of the respondents’ diet in both rural and urban areas. Fried foods were also one of the most eaten groups of products in the studied population, both as snacks and side dishes. Martianto et al. [49] stated that the average consumption of palm oil in Indonesia exceeds 23 g/person/day, with average household usage for cooking being 1–3 times higher. The high daily palm oil consumption makes this product highly feasible as one of the products to be enriched with plant sterols, targeted for Indonesian population. Noodles were also one of the popular products, consumed by most of the respondents in almost every meal, with the average consumption level of 39 g/day, higher in the rural compared to the urban area. The respondents also reported frequent consumption of baked products, including bread, buns, and biscuits, as options for breakfast or snacks, with average consumption of 26 g/day in both areas. 

All the mentioned products are generally used daily, which complies with the requirements for food carriers/vehicles for successful fortification programs. Thus, they all have the potential to be used as successful vehicles of plant sterols enrichment [49] and could be considered as the starter products intended not only for people with diagnosed hypercholesterolemia (first priority), but also for general population, for maintaining healthy blood lipid profile (as a preventive measure). These products, with proposed formulations and information on the recommended consumption level, have been presented in Table 6. 

It should be underlined though that economic aspect of the products’ fortification is very important and should be taken into consideration to ensure that low income groups would benefit from such intervention. Until now, the most cost-effective techniques allow for incorporation of plant sterols and stanols into such food carriers as solid/semi-solid margarines, butter, and liquid vegetable oils. Foods in the solid form such as biscuits or bread could be enriched by plant sterols/stanols through margarine, butter, or vegetable oils as their ingredients.

Incorporation of several enriched products into the diet raises a concern of potential risk of the targeted nutrients/compounds overdosing. However, according to the undertaken estimations, in case if one person would consume more than one product (up to 3–4 products daily) on the recommended consumption level, total daily plant sterols intake would be still in a range generally considered as not posing a significant health risk [50]. Moreover, it should be underlined that the proposed six products are examples of foods which could be the most relevant targets for such fortification, as a starting point for further analysis and decision which of them will be most feasible with regard to the expected impact, safety, cost efficiency, etc.

Biofortification (plant breeding or biotechnology to increase the sterol/stanol production of plants) could be also considered as one of the alternative effective solutions to increase plant sterols intake. It is generally a promising, sustainable, and cost-effective technique of delivering deficient essential nutrients to populations that have limited access to diverse diets and other (micro)nutrient interventions [51]. However, until now the highest priority in such techniques is given to micronutrients that are considered as more important/urgently needed to combat their deficiencies (e.g., selected essential amino acids, and vitamin A). The biofortified food crops, especially legumes, cereals, vegetables, and fruits, have a strong potential to provide sufficient levels of micronutrients to targeted populations [51].

### 4.4. Ongoing Scientific Discussion on Efficiency and Long-Term Safety of Plant Sterols Treatment

As previously mentioned, while many international associations recommend the use of plant sterols and stanols as food additives to reduce blood cholesterol levels [24], other researchers and organizations point to the strong need for long-term, comprehensive studies, allowing to properly assess safety/estimate the potential health risks/side effects of long-term plant sterol treatment before making further decisions on such intervention [25,26,27].

Some recent scientific literature suggests that, like cholesterol, plant sterols may also accumulate in the aortic valve tissue [52,53] and contribute to the development of atherosclerotic lesions [54], especially in subjects with mutations in the ABCG5 and/or ABCG8 gene causing strongly increased absorption/inability to remove plant sterols from the body [55,56]. A potential for plant sterols accumulation in cardiovascular tissue and their causal involvement in the development of atherosclerosis have been a matter of scientific debate over the last decade [27,57,58,59,60,61]. While a number of studies have reported a positive relation between plasma plant sterol concentrations and the risk of atherosclerotic cardiovascular disease [58,62,63,64,65,66], others did not confirm such association [59,67,68] or have demonstrated plant sterols association with reduced cardiovascular risk [69,70,71].

It should be also pointed that on average about 50% of intestinal cholesterol and 2% of plant sterols is absorbed. Increasing the plant sterol intake from 200 mg/day to 2 g/day, as is proclaimed by the providers of plant sterol enriched products, leads to about 30% reduction of the daily cholesterol absorption rate, but also to a tenfold increase of the plant sterol absorption rate. The reduction of cholesterol absorption is undoubtedly a positive effect of plant sterol intake. However, this is partly compensated by an increased cholesterol biosynthesis. The resulting reduction of LDL cholesterol is on average about 10%, but may be much lower in many cases. Different causes of elevated LDL cholesterol should be considered: high cholesterol intake, high absorption rate, high synthesis rate, and low activity of the LDL receptor; also important is the (in)ability to downregulate synthesis when absorption rate is high. Thus, intake of plant sterols as such does not guarantee sufficient reduction of LDL-cholesterol in serum.

Considering all the above-mentioned limitations and concerns, decision about introducing the proposed foods fortified with cholesterol-lowering compounds to the market should be definitely preceded by comprehensive research on the efficacy and safety aspects of long-term plant sterol treatment. The effectiveness and safety of such intervention should be also compared to the known pharmaceutical treatments such as ezetimibe (lowering cholesterol absorption) and statins (lowering cholesterol synthesis) and the combined treatment [72].

## 5. Conclusions

The presented study aimed to analyze, for the first time, the consumption patterns of foods containing plant sterols by general population in Indonesia, and provided up-to-date data about the dietary intake of plant sterols by the Indonesian population. The outcomes and suggestions from this study are expected to open the gate for research on the potential of plant sterols for hypercholesterolemia prevention in Indonesia, and especially in the area of functional food development. The study revealed that the level of plant sterols intake in Bogor reached on average 229.76 mg/day and was not significantly different between urban and rural area. Cereals, vegetables, and fruit products were estimated to be the main food sources of plant sterols in both areas. Interestingly, this research has recorded several indigenous fruits, leaves, vegetables, as well as typical Indonesian foods which have never been reported in the available plant sterol content databases and should be assessed for their plant sterols profiles.

Based on the study findings, a list of several popular surveyed food items, with a good potential to be used as successful vehicles for plant sterols enrichment, was developed as a starting point for further analysis and the decision of which of them would be most feasible with regard to the potential expected impact, safety, cost efficiency, etc. The study outcomes, based on food plant sterol content data available in on-line databases and scientific publications, should be considered to be further confirmed by the laboratory analysis of plant sterol profiles of the particular food products. Such analysis would not only provide much more precise information on the plant sterols content, but also on the cholesterol-lowering potential/effectiveness of particular food items, considering different cholesterol lowering abilities of particular compounds within the plant sterols group.

Although this study undoubtedly provides baseline data on plant sterol intake and draws the directions/paths for potential development of functional foods fortified with plant sterols suitable for the Indonesian needs and taste, it should be underlined that further comprehensive studies are still needed to confirm efficacy and safety of introducing such phytosterol-enriched products into a habitual diet (especially considering widely discussed possible long-term side effects of plant sterol treatment, i.e., their potential contribution to atherosclerosis) before making further decisions on such intervention. It should be also considered that that economic aspect of the products’ fortification is very important and should be taken into consideration to ensure that low income groups would benefit from such intervention. Hereafter, a good collaboration of scientists, governments, and food industries is required to build an effective and safe strategy and tools for hypercholesterolemia prevention in Indonesia.

## Figures and Tables

**Table 1:** Daily food and selected nutrients intake in the studied population of men and women in the rural and urban area of Bogor, Indonesia.

Intake	Rural Area			Urban Area		
	Men	Women	Total	Men	Women	Total
Food (g/day)	1150.34	1055.11	1102.73	1057.2	913.6	985.4
Energy (kcal/day)	1488.65	1485.23	1489.94	1647.50	1491.42	1569.4
Protein (g/day)	51.39	50.49	47.37	50.32	51.59	50.96
Iron (mg/day)	20.00	19.21	17.43	18.25	20.26	19.26
Vitamin A (RE ^1^/day)	222.46	382.40	302.43	346.35	250.52	298.43
Vitamin C (mg/day)	18.07	53.53	35.80	25.46	34.41	29.93

^1^ retinol equivalents.

**Table 2:** Consumptions of foods predicted to contain plant sterols, divided into 12 food groups, in rural and urban area of Bogor.

No	Food Groups	Rural Area					Urban Area				
		Consumption Per Capita (g/day) ^1^				% of Total Consumption	Consumption Per Capita (g/day) ^1^				% of Total Consumption
		Mean ± SD	95% Tile	Min.	Max.		Mean ± SD	95% Tile	Min	Max	
1	Beverages	16.65 ± 27.71	75.35	0.00	152.67	1.88	15.93 ± 29.15	49.51	0.00	250.53	1.75
2	Cereals and Cereal Products	378.01 ± 164.85	692.77	39.02	1046.50	42.66	381.31 ± 132.32	570.91	103.07	759.33	41.98
3	Eggs and Egg Products	8.58 ± 15.60	41.47	0.00	88.00	0.97	10.50 ± 18.16	60.00	0.00	69.33	1.16
4	Fish and Fish Products	1.37 ± 3.63	8.13	0.00	24.43	0.15	2.45 ± 5.55	14.61	0.00	35.20	0.27
5	Fruits and Fruit Products	103.04 ± 106.28	323.93	0.00	504.07	11.63	116.41 ± 116.29	286.77	0.00	630.40	12.81
6	Herbs, Spices, and Condiments	11.91 ± 20.22	56.95	0.00	119.00	1.34	13.88 ± 18.59	50.47	0.00	112.73	1.53
8	Legumes, Legume Products, and Nuts	85.26 ± 63.89	208.73	0.00	385.72	9.62	84.66 ± 72.77	196.85	0.00	386.07	9.32
7	Meat and Meat Products	22.52 ± 32.62	85.58	0.00	214.73	2.54	33.65 ± 36.58	94.66	0.00	237.93	3.70
9	Plant Sterol-Fortified Products	0.04 ± 0.30	0.00	0.00	2.67	0.00	0.03 ± 0.30	0.00	0.00	3.00	0.00
10	Snack Foods	99.07 ± 91.71	253.45	0.00	584.55	11.18	106.91 ± 99.39	266.72	0.00	636.47	11.77
11	Supplements	0.05 ± 0.50	0.00	0.00	5.00	0.01	0.12 ± 0.45	1.37	0.00	2.00	0.01
12	Vegetables and Vegetable Products	159.64 ± 112.93	396.02	0.00	575.37	18.02	142.57 ± 98.44	337.31	20.19	624.18	15.69
	**TOTAL**	**886.14 ± 387.87**	**1759.82**	**205.64**	**2067.78**	**100.00**	**908.43 ± 349.83**	**1549.21**	**344.33**	**2458.76**	**100.00**

^1^ Consumption of foods predicted to contain plant sterols in urban area.

**Table 3:** Compositional data of plant sterol (PS) value in food items consumed by respondents.

Plant Sterol (PS) Data Sources	Number of Food Items Consumed					
	No Total (%)		Rural Area (%)		Urban Area (%)	
Available from secondary data (USDA database and scientific publications)	94	(30.42%)	83	(31.44%)	80	(31.87%)
Commercial product available in the market	1	(0.32%)	1	(0.38%)	1	(0.40%)
Calculation of recipe	162	(52.43%)	142	(53.79%)	127	(50.60%)
PS content set as zero	27	(8.74%)	19	(7.20%)	22	(8.76%)
Data not available (thus PS content set as zero)	25	(8.09%)	19	(7.20%)	21	(8.37%)
**Total**	**309**	**(100.00%)**	**264**	(100.00%)	**251**	(100.00%)

**Table 4:** The contribution of each of the food groups to plant sterol intake in the rural and urban population (mg/person/day).

No	Food Groups	Rural Area			Urban Area		
		Male	Female	Total	Male	Female	Total
1	Beverages	0.01 ± 0.10	0.00 ± 0.00	0.01 ± 0.07	0.00 ± 0.00	0.00 ± 0.00	0.00 ± 0.00
2	Cereals and Cereal Products	82.30 ± 37.39	85.37 ± 46.38	83.84 ± 41.94	95.55 ± 34.05	79.89 ± 31.38	87.72 ± 33.51
3	Eggs and Egg Products	0.61 ± 1.19	1.05 ± 1.69	0.83 ± 1.47	1.70 ± 3.15	1.07 ± 2.49	1.39 ± 2.84
4	Fish and Fish Products	0.17 ± 0.39	0.51 ± 2.39	0.34 ± 1.72	0.56 ± 1.08	0.58 ± 1.64	0.57 ± 1.38
5	Fruits and Fruit Products	11.59 ± 13.33	12.94 ± 13.85	12.26 ± 13.54	12.08 ± 13.13	17.44 ± 28.19	14.76 ± 22.04
6	Herbs, Spices and Condiments	3.53 ± 7.60	5.62 ± 12.18	4.58 ± 10.15	4.61 ± 6.38	8.18 ± 10.55	6.39 ± 8.86
8	Legumes and Legume Products	53.51 ± 48.90	55.73 ± 45.44	54.62 ± 46.98	55.23 ± 44.07	56.00 ± 59.45	55.62 ± 52.06
7	Meat and Meat Products	4.00 ± 11.45	4.63 ± 7.30	4.31 ± 9.56	4.47 ± 4.24	4.36 ± 5.50	4.42 ± 4.89
9	Phytosterol Fortified Products	0.05 ± 0.34	0.02 ± 0.17	0.04 ± 0.26	0.00 ± 0.00	0.05 ± 0.38	0.03 ± 0.27
10	Snack Foods	40.77 ± 50.00	27.83 ± 22.19	34.30 ± 39.03	33.28 ± 31.39	39.42 ± 37.79	36.35 ± 34.70
11	Supplements	0.00 ± 0.00	0.00 ± 0.00	0.00 ± 0.00	0.00 ± 0.00	0.00 ± 0.00	0.00 ± 0.00
12	Vegetables and Vegetable Products	26.87 ± 22.23	30.49 ± 20.86	28.68 ± 21.52	26.11 ± 26.63	30.85 ± 22.75	28.48 ± 24.76
**TOTAL**		**223.41 ± 120.89**	**224.19 ± 97.64**	**223.80 ± 109.33**	**233.61 ± 93.45**	**237.84 ± 115.45**	**235.73 ± 104.52**

**Table 5:** List of products fortified with plant sterols available in the Bogor market.

No	Product Type	Brand	Plant Sterols Type	Plant Sterols Concentration per Serving
1	Milk Powder	Tropicana Slim Non-Fat Fitosterol	Phytosterols	20.1 mg
2	Smoothie	Nutrive Benecol	Plant stanol ester	1.7 g
3	Supplement	GNC Triple Fish With Phytosterols	Phytosterols	800 mg
4	Supplement	Hemaviton Cardio	Phytosterols	400 mg
5	Supplement	Natrol Cholesterol Balance	Beta Sitosterol	440 mg
6	Supplement	Nature’s Plus Thermo Tropic	Phytosterols	100 mg
7	Supplement	Nutrimax Prost Care	Beta Sitosterol	150 mg
8	Supplement	TRA Complex	Beta Sitosterol	40 mg

**Table 6:** Examples of food products to be enriched with plant sterols or plant sterol esters, target concentrations of plant sterols, and recommended consumption levels.

Product Category	Common Packing Size	Plant Sterols/Plant Sterol Esters Concentration	Recommended Daily Consumption Level	Total Daily Intake of Plant Sterols
Bread	60 g	1.5 g/100 g	1 × 70–80 g/day	1.2–1.5 g
Biscuits	100 g	3.3 g/100 g	1–2 × 15–20 g/day	1.2–1.5 g
Noodles/Pasta	65 g	1.5–1.7 g/100 g	1 × 60–80 g/day	1.2–1.5 g
Fat spreads /margarines	200 g	4.8–5.0 g/100 g	2–3 × 10 g/day	1.2–1.5 g
Spicy sauces	140 g	5.0–9.0 g/100 mL	1–2 × 10 g/day	1.1–1.4 g
Palm Oil	1000 g	3.9–4.4 g/100 mL	1–2 × 12 g/day	1.2–1.6 g

## Data Availability

Data will be made available upon request by author Prof. Nuri Andarwulan.

## Supplementary Materials

The following are available online at https://www.mdpi.com/2072-6643/13/2/452/s1, Table S1: Respondents characteristics, Table S2: Average of plant sterols content in each food group and sub-group consumed by all respondents, Table S3: Food items with no relevant data on plant sterols content and their percentage of total consumption in rural and urban area, Table S4: Plant sterols (PS) content in each food group, its total consumption, and contribution to overall plant sterols intake by rural respondents, Table S5: Plant sterols (PS) content in each food group, its total consumption, and contribution to overall plant sterols intake by urban respondents, Table S6: Plant sterols (PS) content in every food item consumed by the respondents, Table S7: Example of plant sterols (PS) content calculation based on product or meal recipe, Table S8: Plant sterols (PS) content based on calculation from the product or meal recipe, Figure S1: Respondents selection flowchart, Figure S2: The average plant sterols content in each of the food groups.
