# Supplementary material for: Cross-Sectional Study of Plant Sterols Intake as a Basis for Designing Appropriate Plant Sterol-Enriched Food in Indonesia
Source: Nutrients. 2021 Jan 29;13(2):452. doi: 10.3390/nu13020452 (PMC7912629; doi:10.3390/nu13020452)
Supplement: Supplementary file 1 [file nutrients-13-00452-s001.pdf]

Supplementary Materials for the Article

Cross Sectional Study of Plant Sterols Intake as a Basis for Designing Appropriate Plant Sterols Enriched Food in Indonesia

Drajat Martianto [1,2], Atikah Bararah [3], Nuri Andarwulan [1,3] and Dominika Średnicka-Tober [4,*]

[1] Southeast Asian Food and Agricultural Science and Technology (SEAFAST) Center, IPB University, IPB Darmaga Campus, Bogor 16680, West Java, Indonesia; drajat_martianto@yahoo.com (D.M.), andarwulan@yahoo.com (N.A.)
[2] Department of Community Nutrition, Faculty of Human Ecology, IPB University, IPB Darmaga Campus, Bogor 16680, West Java, Indonesia; drajat_martianto@yahoo.com (D.M.)
[3] Department of Food Science and Technology, Faculty of Agricultural Technology, IPB University, P.O. Box 220, IPB Darmaga Campus, Bogor 16680, West Java, Indonesia; atikah.bararah@gmail.com (A.B.), andarwulan@yahoo.com (N.A.)
[4] Department of Functional and Organic Food, Institute of Human Nutrition Sciences, Warsaw University of Life Sciences, Nowoursynowska 159c, 02-776 Warsaw, Poland; dominika_srednicka_tober@sggw.edu.pl (D.Ś.-T.)
* Correspondence: dominika_srednicka_tober@sggw.edu.pl; Tel.: +48225937035 (D.Ś.-T.).

List of Tables

Table S1. Respondents characteristics. 3
Table S2. Average of plant sterols content in each food group and sub-group consumed by all respondents. 5
Table S3. Food items with no relevant data on plant sterols content and their percentage of total consumption in rural and urban area. 7
Table S4. Plant sterols (PS) content in each food group, its total consumption, and contribution to overall plant sterols intake by rural respondents. 9
Table S5. Plant sterols (PS) content in each food group, its total consumption, and contribution to overall plant sterols intake by urban respondents.. 12
Table S6. Plant sterols (PS) content in every food item consumed by the respondents. 15
Table S7. Example of plant sterols (PS) content calculation based on product or meal recipe. 34
Table S8. Plant sterols (PS) content based on calculation from the product or meal recipe. 35

List of Figures

Figure S1. Respondents selection flowchart. 2
Figure S2. The average plant sterols content in each of the food groups. 8

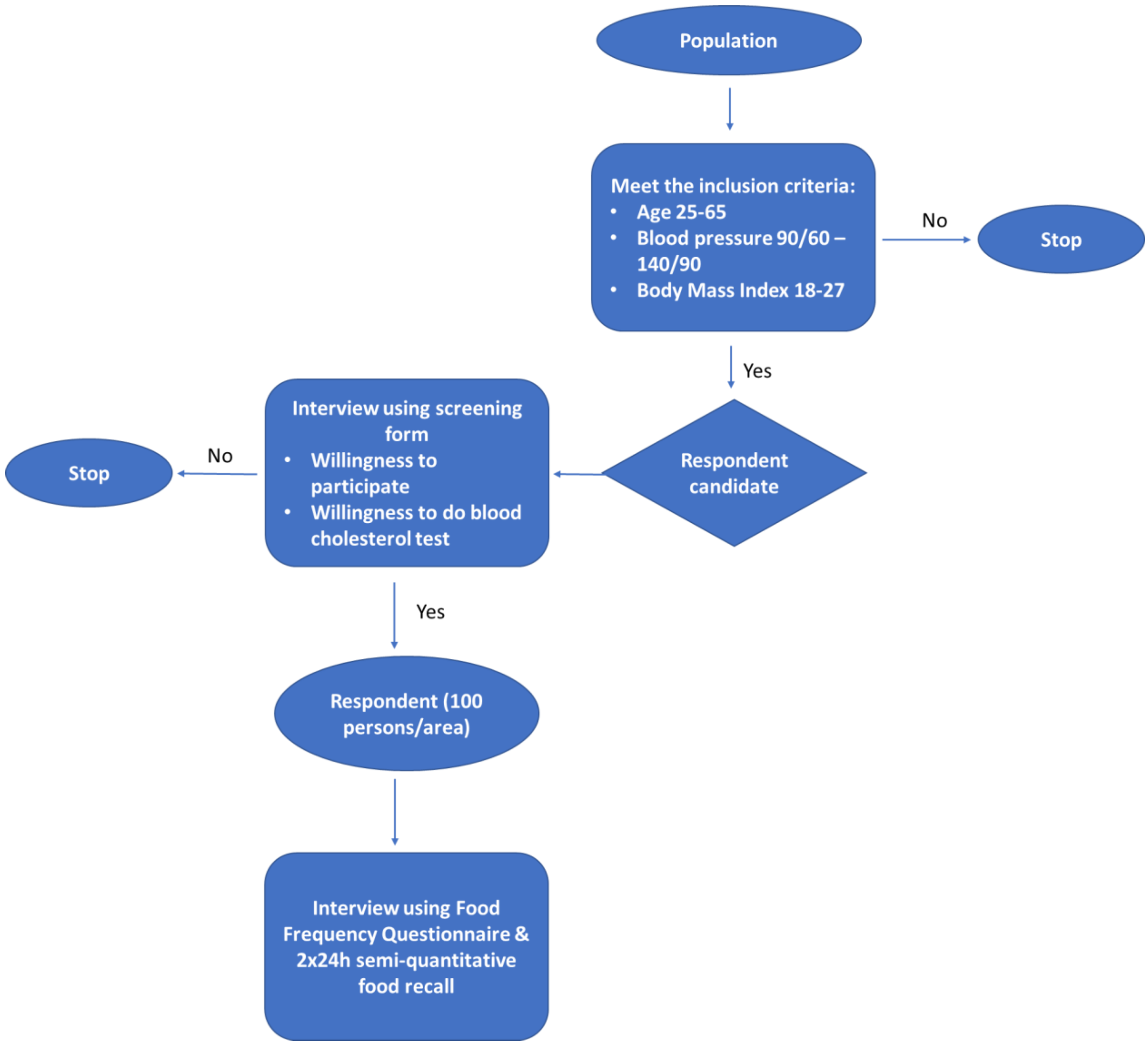

Figure S1. Respondents selection flowchart.

Table S1. Respondents characteristics.

Characteristics	Rural Area						Urban Area					
	Male		Female		Total		Male		Female		Total	
	n	%	n	%	n	%	n	%	n	%	n	%
Age Group												
25 - 35 years	23	46	22	44	45	45	13	26	23	46	36	36
36 - 45 years	7	14	12	24	19	19	15	30	9	18	24	24
46 - 55 years	13	26	11	22	24	24	10	20	9	18	19	19
56 - 65 years	7	14	5	10	12	12	12	24	9	18	21	21
**Total**	**50**	**100**	**50**	**100**	**100**	**100**	**50**	**100**	**50**	**100**	**100**	**100**
Education Level												
Uneducated	3	6	7	14	10	10	1	2	3	6	4	4
Primary School	25	50	22	44	47	47	10	20	14	28	24	24
Junior High School	10	20	12	24	22	22	7	14	15	30	22	22
Senior High School	8	16	8	16	16	16	28	56	12	24	40	40
Diploma	1	2	0	0	1	1	3	6	3	6	6	6
College	2	4	1	2	3	3	1	2	3	6	4	4
Bachelor Degree	1	2	0	0	1	1	0	0	0	0	0	0
**Total**	**50**	**100**	**50**	**100**	**100**	**100**	**50**	**100**	**50**	**100**	**100**	**100**
Type of Job												
Government Official	4	8	0	0	4	4	3	6	3	6	6	6
Private Official	4	8	0	0	4	4	9	18	0	0	9	9
Business	12	24	0	0	12	12	14	28	1	2	15	15
Farmer	2	4	1	2	3	3	0	0	0	0	0	0
Labor	20	40	4	8	24	24	11	22	0	0	11	11
Teacher	0	0	0	0	0	0	1	2	2	4	3	3
Housewife	0	0	40	80	40	40	0	0	38	76	38	38
Seller	2	4	5	10	7	7	3	6	4	8	7	7
Others	6	12	0	0	6	6	6	12	2	4	8	8
No Job	0	0	0	0	0	0	3	6	0	0	3	3
**Total**	**50**	**100**	**50**	**100**	**100**	**100**	**50**	**100**	**50**	**100**	**100**	**100**
Household Income (Rp/month)												
< 1000 000	19	38	24	48	43	43	10	20	26	52	36	36
1 000 000 – 2 000 000	20	40	18	36	38	38	10	20	15	30	25	25
2 000 000 – 4 000 000	7	14	8	16	15	15	23	46	4	8	27	27
4 000 000 – 8 000 000	4	8	0	0	4	4	6	12	5	10	11	11
> 8 000 000	0	0	0	0	0	0	1	2	0	0	1	1
**Total**	**50**	**100**	**50**	**100**	**100**	**100**	**50**	**100**	**50**	**100**	**100**	**100**
**Mean ± SD**	**1 294 000 ± 958 852**						**2 030 500 ± 2 077 803**					
No of Household Members												
Small (≤ 4 persons)	22	44	28	56	50	50	28	56	34	68	62	62
Medium (5-6 persons)	19	38	17	34	36	36	18	36	13	26	31	31
Large (≥ 7 persons)	9	18	5	10	14	14	4	8	3	6	7	7
**Total**	**50**	**100**	**50**	**100**	**100**	**100**	**50**	**100**	**50**	**100**	**100**	**100**
**Mean**	**4 - 5 persons**						**4 - 5 persons**					

**Nutritional Status**												
**Underweight (BMI 16-18.5)**	6	12	4	8	10	10	3	6	3	6	6	6
**Normal (BMI 18.5-24.9)**	43	86	39	78	82	82	38	76	36	72	74	74
**Overweight (BMI 25.0-29.0)**	1	2	7	14	8	8	9	18	11	22	20	20
**Total**	**50**	**100**	**50**	**100**	**100**	**100**	**50**	**100**	**50**	**100**	**100**	**100**
**Mean ± SD**	**21.62 ± 2.30**						**22.78 ± 2.63**					
**Blood pressure**												
**Hypertension**	0	0	1	2	1	1	4	8	5	10	9	9
**Pre-hypertension**	15	30	12	24	27	27	13	26	11	22	24	24
**Normal**	35	70	37	74	72	72	33	66	34	68	67	67
**Total**	**50**	**100**	**50**	**100**	**100**	**100**	**50**	**100**	**50**	**100**	**100**	**100**
**Mean ± SD**	**118.10/77.75 ± 10.22/6.94**						**119.20/79.30 ± 12.20/7.42**					

Table S2. Average of plant sterols content in each food group and sub-group consumed by all respondents.

No	Food Groups and Sub-Groups	Plant sterols content (mg/100g of food)						
		**Mean**	**±**	**S.D.**	**95% tile**	**Min**	**-**	**Max**
**1**	**Beverages**	**0.19**	**±**	**0.69**	**1.00**	**0.00**	**-**	**2.49**
**2**	**Cereals and cereal products**	**32.24**	**±**	**20.17**	**50.62**	**0.00**	**-**	**120.19**
	cereal product, brown rice based	26.80	±	0.00	26.80	26.80	-	26.80
	cereal product, corn based	32.45	±	12.51	47.34	22.13	-	50.67
	cereal product, oat based	44.00	±	0.00	44.00	44.00	-	44.00
	cereal product, processed	20.61	±	0.00	20.61	20.61	-	20.61
	cereal product, rice based	32.20	±	9.46	42.52	22.50	-	43.71
	cereal product, rice flour based	24.36	±	2.62	26.03	22.50	-	26.21
	cereal product, wheat flour based	29.95	±	21.66	46.55	0.00	-	46.86
	composite food, rice based	43.92	±	43.23	103.25	16.83	-	120.19
	composite food, rice flour based	31.81	±	0.00	31.81	31.81	-	31.81
	composite food, wheat flour based	18.85	±	4.74	24.49	14.98	-	25.45
	dishes with peanut sauce, rice based	41.41	±	4.95	44.56	37.91	-	44.91
	dishes with peanut sauce, wheat flour based	50.56	±	0.00	50.56	50.56	-	50.56
**3**	**Eggs and egg products**	**6.51**	**±**	**4.44**	**9.70**	**0.00**	**-**	**10.00**
	egg with chili and spices sauce	8.02	±	0.00	8.02	8.02	-	8.02
	egg, boiled	0.00	±	0.00	0.00	0.00	-	0.00
	fried dishes, egg	9.00	±	1.41	9.90	8.00	-	10.00
**4**	**Fish and fish products**	**16.66**	**±**	**5.06**	**19.74**	**6.45**	**-**	**20.20**
**5**	**Fruits and fruit products**	**22.61**	**±**	**24.72**	**79.85**	**2.00**	**-**	**83.00**
	composite food, mix fruit	15.99	±	9.41	21.97	9.33	-	22.64
	dishes with peanut sauce, fruit	n.a	±	n.a	n.a	n.a	-	n.a
	dishes with peanut sauce, mix fruits	59.17	±	0.00	59.17	59.17	-	59.17
	fruits, processed, dried	62.00	±	0.00	62.00	62.00	-	62.00
	fruits, raw	20.31	±	25.05	83.00	2.00	-	83.00
	jam and jelly	12.00	±	0.00	12.00	12.00	-	12.00
**6**	**Herbs, spices and condiments**	**40.19**	**±**	**62.91**	**162.23**	**0.00**	**-**	**200.11**
	condiments	60.29	±	69.35	175.76	0.00	-	200.11
	traditional herbal drinks	0.00	±	0.00	0.00	0.00	-	0.00
**7**	**Legumes and legume products**	**92.28**	**±**	**111.79**	**220.00**	**0.00**	**-**	**534.00**
	composite food, oncom based	0.85	±	1.21	1.62	0.00	-	1.71
	composite food, peanut based	131.76	±	0.00	131.76	131.76	-	131.76
	composite food, red bean soup	127.00	±	0.00	127.00	127.00	-	127.00
	composite food, tempeh based	103.54	±	49.91	145.62	24.10	-	148.23
	composite food, tofu based	13.81	±	14.76	36.28	0.82	-	45.64
	legumes, mung bean based	11.16	±	6.02	14.98	6.90	-	15.41
	legumes, other (cashew, fava, etc.)	195.20	±	196.50	458.80	22.00	-	534.00
	legumes, peanut based	159.50	±	78.64	220.00	50.00	-	220.00
	legumes, soy based	5.56	±	0.00	5.56	5.56	-	5.56
**8**	**Meats and meat product**	**17.01**	**±**	**19.01**	**62.10**	**2.57**	**-**	**63.07**
	composite food, meat based	7.08	±	3.47	10.74	3.77	-	11.07
	composite food, poultry based	7.14	±	4.23	10.63	2.57	-	10.93

No	Food Groups and Sub-Groups	Plant sterols content (mg/100g of food)						
		Mean	±	S.D.	95% tile	Min	-	Max
	dishes with peanut sauce, meat or poultry based	62.37	±	0.98	63.00	61.68	-	63.07
	fried dishes, meat based	5.55	±	0.00	5.55	5.55	-	5.55
	fried dishes, poultry based	15.01	±	2.91	16.97	9.95	-	16.97
**9**	**Phytosterol fortified products**	**88.90**	**±**	**0.00**	**88.90**	**88.90**	**-**	**88.90**
**10**	**Snack food**	**29.45**	**±**	**30.85**	**101.20**	**0.00**	**-**	**133.53**
	biscuits	33.80	±	8.50	45.20	30.00	-	49.00
	bread	0.00	±	0.00	0.00	0.00	-	0.00
	dessert	5.50	±	7.78	10.45	0.00	-	11.00
	fried snacks, plantain based	22.74	±	1.35	23.59	21.78	-	23.69
	fried snacks, rice flour based	33.85	±	18.52	54.45	6.87	-	59.16
	fried snacks, soy based	53.60	±	58.96	126.87	9.74	-	133.53
	fried snacks, tuber based	16.57	±	11.69	36.23	0.00	-	38.95
	fried snacks, wheat flour based	44.06	±	39.05	121.09	10.09	-	131.74
	other bakery wares	35.73	±	28.80	71.80	0.00	-	73.96
	sweets	73.33	±	24.83	97.70	59.00	-	102.00
	traditional cake and snacks	22.33	±	19.98	53.60	0.00	-	68.45
**11**	**Supplements**	**0.00**	**±**	**0.00**	**0.00**	**0.00**	**-**	**0.00**
**12**	**Vegetables and vegetable products**	**19.90**	**±**	**25.37**	**44.22**	**0.00**	**-**	**175.00**
	composite food, mix vegetables soup	21.70	±	14.89	43.53	6.61	-	44.83
	composite food, mix vegetables, raw or steamed	22.92	±	21.33	36.49	7.83	-	38.00
	composite food, mix vegetables, stir fried	10.83	±	0.00	10.83	10.83	-	10.83
	dishes with peanut sauce, leafy or stem vegetable	43.72	±	0.00	43.72	43.72	-	43.72
	dishes with peanut sauce, mix vegetable	45.70	±	13.68	59.06	34.80	-	61.04
	vegetables, processed, fermented	0.00	±	0.00	0.00	0.00	-	0.00
	vegetables, processed, fried	24.66	±	16.63	42.11	5.49	-	43.50
	vegetables, processed, steamed or boiled	9.88	±	6.84	20.63	0.44	-	21.96
	vegetables, processed, stir fried	23.81	±	38.08	53.14	1.74	-	175.00
	vegetables, raw	17.98	±	26.14	55.00	0.00	-	106.00

Table S3. Food items with no relevant data on plant sterols content and their percentage of total consumption in rural and urban area.

No	Food Name	Indonesian Name	Food Group	% contribution in rural area	% contribution in urban area
1	Carambolas, raw	Belimbing	fruits and fruit products	0.000	0.044
2	Custard apple, soursops, raw	Sirsak	fruits and fruit products	0.023	0.008
3	Custard apple with sugar	Rujak sirsak	fruits and fruit products	0.051	0.036
4	Durian	Durian	fruits and fruit products	0.023	0.029
5	Guavas, raw	Jambu biji	fruits and fruit products	0.270	0.360
6	Guavas, raw, with sugar	Rujak jambu	fruits and fruit products	0.008	0.000
7	Jackfruits, raw	Nangka	fruits and fruit products	0.060	0.013
8	Longans, raw	Leci	fruits and fruit products	0.004	0.017
9	Mango, served with salt and dip peanut sauce	Rujak mangga	fruits and fruit products	0.000	0.065
10	Mangos, raw	Mangga	fruits and fruit products	0.860	1.422
11	Papayas, raw	Pepaya	fruits and fruit products	2.970	2.809
12	Rambutans, raw	Rambutan	fruits and fruit products	0.020	0.055
13	Rose apples, raw	Jambu air	fruits and fruit products	0.020	0.000
14	Sapodillas, raw	Sawo	fruits and fruit products	0.030	0.000
15	Snakeskin, raw	Salak	fruits and fruit products	0.170	0.026
16	Betel leaf	Daun sirih	herbs, spices & condiments	0.000	0.001
17	*Zingiber zerumbet L*, leaves	Lempuyang	herbs, spices & condiments	0.000	0.004
18	*Andrographis paniculata*, leaves	Sambiloto	herbs, spices & condiments	0.000	0.002
19	Soybean paste	Tauco	herbs, spices & condiments	0.015	0.004
20	Traditional cake, cassava, mashed, steamed (Getuk)	Getuk	Snack food	0.040	0.000
21	Papaya leaves, steamed	Daun papaya	Vegetables products	0.004	0.003
22	Allspice, raw	Daun poh-pohan	Vegetables products	0.003	0.001
23	Cassava leaves, steamed	Daun singkong	Vegetables products	0.030	0.036
24	Cassava, steamed	Singkong	Vegetables products	0.920	0.674
25	Dogfruit, raw or stir fried	Jengkol	Vegetables products	0.000	0.011
		**Total**		**5.521**	**5.620**

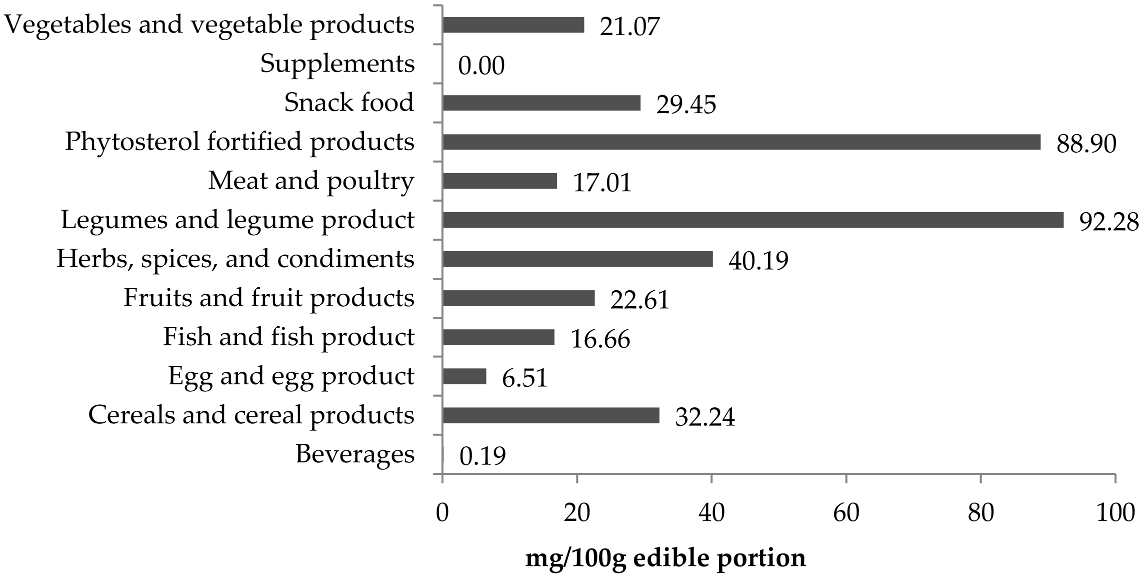

Figure S2. The average plant sterols content in each of the food groups.

Table S4. Plant sterols (PS) content in each food group, its total consumption, and contribution to overall plant sterols intake by rural respondents.

No	Food Groups	Plant sterols content (mg/100g of food)			Daily consumption (g/person/day)			% Total Consumption	Plant sterols intake (mg/person/day)			% Total Intake	No. of Eater (N=100)	% of Eater (N=100)
		Mean	±	S.D.	Mean	±	S.D.		Mean	±	S.D.			
1	**Beverages**	**0.21**	±	**0.72**	**16.65**	±	**27.71**	**1.88**	**0.01**	±	**0.07**	**0.00**	59	59%
2	**Cereals and Cereal Products**	**30.75**	±	**21.86**	**378.01**	±	**164.85**	**42.66**	**83.84**	±	**41.94**	**37.46**	100	100%
	cereal product, brown rice based	26.80	±	0.00	4.83	±	48.00	0.54	1.29	±	12.86	0.58	2	2%
	cereal product, corn based	26.38	±	3.68	13.49	±	80.08	1.52	3.83	±	22.82	1.71	23	23%
	cereal product, oat based	44.00	±	0.00	3.28	±	32.00	0.37	1.22	±	11.88	0.55	2	2%
	cereal product, processed	20.61	±	0.00	28.58	±	45.35	3.23	1.67	±	3.08	0.74	84	84%
	cereal product, rice based	32.20	±	9.46	273.06	±	106.24	30.81	61.61	±	23.89	27.53	99	99%
	cereal product, rice flour based	13.11	±	18.53	4.89	±	7.32	0.55	1.28	±	1.97	0.57	56	56%
	cereal product, wheat flour based	14.05	±	19.87	1.05	±	7.17	0.12	0.19	±	1.57	0.09	3	3%
	composite food, rice based	43.92	±	43.23	14.41	±	34.42	1.63	3.18	±	7.26	1.42	35	35%
	composite food, rice flour based	31.81	±	0.00	0.05	±	0.50	0.01	0.02	±	0.16	0.01	1	1%
	composite food, wheat flour based	20.21	±	7.40	2.51	±	13.86	0.28	0.35	±	1.90	0.16	6	6%
	dishes with peanut sauce, rice based	41.41	±	4.95	11.31	±	25.23	1.28	4.88	±	11.08	2.18	36	36%
	dishes with peanut sauce, wheat flour based	50.56	±	0.00	20.54	±	36.61	2.32	4.31	±	9.99	1.92	48	48%
3	**Eggs and Egg Products**	**9.00**	±	**1.41**	**8.58**	±	**15.60**	**0.97**	**0.83**	±	**1.47**	**0.37**	34	34%
	fried dishes, egg	9.00	±	1.41	8.58	±	15.60	0.97	0.83	±	1.47	0.37	34	34%
4	**Fish and Fish Products**	**15.83**	±	**6.31**	**1.37**	±	**3.63**	**0.15**	**0.34**	±	**1.72**	**0.15**	23	23%
5	**Fruits and Fruit Products**	**24.50**	±	**26.63**	**103.04**	±	**106.28**	**11.63**	**12.26**	±	**13.54**	**5.48**	98	98%
	composite food, mix fruit	15.99	±	9.41	1.61	±	13.83	0.18	0.33	±	3.12	0.15	4	4%
	dishes with peanut sauce, mix fruits	59.17	±	0.00	3.01	±	8.83	0.34	1.79	±	5.24	0.80	15	15%
	fruits, processed, dried	62.00	±	0.00	0.36	±	3.03	0.04	0.22	±	1.88	0.10	3	3%
	fruits, raw	21.05	±	26.39	98.06	±	103.36	11.07	9.92	±	11.18	4.43	97	97%
6	**Herbs, Spices and Condiments**	**36.13**	±	**63.21**	**11.91**	±	**20.22**	**1.34**	**4.58**	±	**10.15**	**2.05**	91	91%
	condiments	56.21	±	72.28	8.33	±	11.39	0.94	4.57	±	10.15	2.04	90	90%

No	Food Groups	Plant sterols content (mg/100g of food)			Daily consumption (g/person/day)			% Total Consumption	Plant sterols intake (mg/person/day)			% Total Intake	No. of Eater (N=100)	% of Eater (N=100)
		Mean	±	S.D.	Mean	±	S.D.		Mean	±	S.D.			
	traditional herbal drinks	0.00	±	0.00	3.59	±	15.17	0.40	0.00	±	0.02	0.00	13	13%
**7**	**Meat and meat Products**	**17.72**	±	**21.33**	**22.52**	±	**32.62**	**2.54**	**4.31**	±	**9.56**	**1.93**	73	73%
	composite food, meat based	7.08	±	3.47	4.30	±	12.66	0.49	0.30	±	1.17	0.14	25	25%
	composite food, poultry based	7.14	±	4.23	1.88	±	4.53	0.21	0.17	±	0.41	0.08	20	20%
	dishes with peanut sauce, meat or poultry based	62.37	±	0.98	2.90	±	12.78	0.33	1.72	±	7.91	0.77	12	12%
	fried dishes, meat based	5.55	±	0.00	0.03	±	0.33	0.00	0.00	±	0.02	0.00	1	1%
	fried dishes, poultry based	16.27	±	0.99	13.40	±	24.84	1.51	2.12	±	3.90	0.95	58	58%
**8**	**Legumes and Legume Products**	**88.72**	±	**110.58**	**85.26**	±	**63.89**	**9.62**	**54.62**	±	**46.98**	**24.41**	99	99%
	composite food, oncom based	0.85	±	1.21	4.32	±	8.17	0.49	0.08	±	0.16	0.03	58	58%
	composite food, peanut based	131.76	±	0.00	0.04	±	0.28	0.00	0.05	±	0.36	0.02	2	2%
	composite food, red bean soup	127.00	±	0.00	0.15	±	1.51	0.02	0.10	±	1.04	0.05	1	1%
	composite food, tempeh based	103.54	±	49.91	30.51	±	25.81	3.44	40.52	±	34.61	18.11	96	96%
	composite food, tofu based	13.81	±	14.76	21.96	±	19.98	2.48	1.83	±	1.79	0.82	86	86%
	legumes, mung bean based	11.16	±	6.02	23.38	±	44.22	2.64	2.80	±	5.64	1.25	50	50%
	legumes, other (cashew, fava, etc.)	238.50	±	197.44	0.71	±	3.48	0.08	2.52	±	16.33	1.13	12	12%
	legumes, peanut based	144.38	±	81.98	3.08	±	10.46	0.35	6.61	±	21.69	2.95	31	31%
	legumes, soy based	27.78	±	31.42	1.12	±	6.86	0.13	0.10	±	0.56	0.05	6	6%
**9**	**Phytosterol Fortified Products**	**88.90**	±	**0.00**	**0.04**	±	**0.30**	**0.00**	**0.04**	±	**0.26**	**0.02**	2	2%
**10**	**Snack Foods**	**28.39**	±	**28.71**	**99.07**	±	**91.71**	**11.18**	**34.30**	±	**39.03**	**15.33**	98	98%
	biscuits	33.80	±	8.50	1.50	±	3.16	0.17	0.54	±	1.16	0.24	34	34%
	bread	0.00	±	0.00	7.93	±	14.21	0.89	0.10	±	0.29	0.05	53	53%
	dessert	5.50	±	7.78	0.15	±	1.03	0.02	0.01	±	0.09	0.00	2	2%
	fried snacks, plantain based	22.74	±	1.35	7.83	±	49.40	0.88	2.60	±	17.70	1.16	18	18%
	fried snacks, rice flour based	33.86	±	21.38	1.30	±	4.38	0.15	0.16	±	0.64	0.07	16	16%
	fried snacks, soy based	64.57	±	61.92	24.97	±	30.59	2.82	17.14	±	24.52	7.66	81	81%
	fried snacks, tuber based	16.57	±	11.69	16.00	±	28.86	1.81	3.32	±	7.72	1.48	62	62%
	fried snacks, wheat flour based	37.85	±	34.19	32.83	±	42.87	3.70	7.55	±	10.52	3.38	86	86%

No	Food Groups	Plant sterols content (mg/100g of food)			Daily consumption (g/person/day)			% Total Consumption	Plant sterols intake (mg/person/day)			% Total Intake	No. of Eater (N=100)	% of Eater (N=100)
		Mean	±	S.D.	Mean	±	S.D.		Mean	±	S.D.			
	other bakery wares	35.73	±	28.80	4.37	±	7.08	0.49	2.27	±	3.68	1.01	49	49%
	sweets	59.00	±	0.00	0.38	±	1.33	0.04	0.22	±	0.79	0.10	13	13%
	traditional cake and snacks	23.27	±	21.15	1.83	±	5.71	0.21	0.38	±	1.26	0.17	17	17%
**11**	**Supplements**	**0.00**	±	**0.00**	**0.05**	±	**0.50**	**0.01**	**0.00**	±	**0.00**	**0.00**	2	2%
**12**	**Vegetables and Vegetable Products**	**17.24**	±	**17.27**	**159.64**	±	**112.93**	**18.02**	**28.68**	±	**21.52**	**12.82**	99	99%
	composite food, mix vegetables soup	21.70	±	14.89	25.91	±	23.83	2.92	7.80	±	8.09	3.48	87	87%
	composite food, mix vegetables, steamed	7.83	±	0.00	0.02	±	0.24	0.00	0.09	±	0.38	0.04	1	1%
	composite food, mix vegetables, stir fried	10.83	±	0.00	1.21	±	4.28	0.14	0.05	±	0.30	0.02	13	13%
	dishes with peanut sauce, mix vegetable	45.70	±	13.68	22.98	±	37.56	2.59	8.77	±	14.40	3.92	65	65%
	vegetables, processed, fermented	0.00	±	0.00	0.12	±	0.84	0.01	0.00	±	0.00	0.00	2	2%
	vegetables, processed, fried	14.40	±	12.13	6.86	±	12.31	0.77	0.61	±	0.91	0.27	49	49%
	vegetables, processed, steamed	12.00	±	0.00	12.09	±	23.76	1.36	1.45	±	2.85	0.65	48	48%
	vegetables, processed, steamed or boiled	9.74	±	7.05	43.75	±	51.90	4.94	2.50	±	3.68	1.12	86	86%
	vegetables, processed, stir fried	14.76	±	11.36	26.44	±	34.96	2.98	4.71	±	5.72	2.11	89	89%
	vegetables, raw	24.79	±	29.05	20.26	±	39.75	2.29	2.71	±	5.21	1.21	50	50%
	**TOTAL**	**30.58**	±	**49.86**	**886.14**	±	**387.87**	**100.00**	**223.80**	±	**109.33**	**100.00**	100	100%

Table S5. Plant sterols (PS) content in each food group, its total consumption, and contribution to overall plant sterols intake by urban respondents..

No	Food Groups	PS content (mg/100g of food)			Food Consumption (g/person/day)			% Total Consumption	PS Intake (mg/person/day)			% Total Intake	No. of Eater (N=100)	% of Eater (N=100)
		Mean	±	S.D.	Mean	±	S.D.		Mean	±	S.D.			
1	**Beverages**	**0.00**	±	**0.00**	**15.93**	±	**29.15**	**1.75**	**0.00**	±	**0.00**	**0.00**	**72**	72%
2	**Cereals and cereal products**	**34.32**	±	**19.88**	**381.31**	±	**132.32**	**41.98**	**87.72**	±	**33.51**	**37.21**	**100**	100%
	cereal product, brown rice based	26.80	±	0.00	0.01	±	0.12	0.00	0.00	±	0.03	0.00	1	1%
	cereal product, corn based	32.45	±	12.51	2.28	±	6.95	0.25	0.74	±	2.67	0.32	26	26%
	cereal product, oat based	44.00	±	0.00	0.89	±	5.82	0.10	0.01	±	0.10	0.00	5	5%
	cereal product, processed	20.61	±	0.00	14.24	±	19.49	1.57	0.72	±	0.93	0.31	68	68%
	cereal product, rice based	31.12	±	8.54	308.32	±	114.53	33.94	69.52	±	25.85	29.49	100	100%
	cereal product, rice flour based	24.36	±	2.62	5.74	±	14.35	0.63	1.54	±	3.91	0.65	40	40%
	cereal product, wheat flour based	45.84	±	1.44	0.58	±	3.33	0.06	0.26	±	1.51	0.11	3	3%
	composite food, rice based	60.86	±	51.55	20.37	±	45.86	2.24	4.86	±	13.24	2.06	42	42%
	composite food, rice flour based	31.81	±	0.00	0.08	±	0.75	0.01	0.02	±	0.18	0.01	1	1%
	composite food, wheat flour based	18.85	±	4.74	1.94	±	7.29	0.21	0.35	±	1.40	0.15	11	11%
	dishes with peanut sauce, rice based	41.41	±	4.95	17.66	±	35.23	1.94	6.74	±	13.70	2.86	35	35%
	dishes with peanut sauce, wheat flour based	50.56	±	0.00	9.20	±	17.81	1.01	2.94	±	5.64	1.25	38	38%
3	**Eggs and egg products**	**12.60**	±	**6.95**	**10.50**	±	**18.16**	**1.16**	**1.39**	±	**2.84**	**0.59**	**40**	40%
	Egg in chili and spices sauce	8.02	±	0.00	0.45	±	3.10	0.05	0.04	±	0.25	0.02	3	3%
	egg, boiled	0.00	±	0.00	0.06	±	0.55	0.01	0.00	±	0.00	0.00	1	1%
	fried dishes, egg	9.00	±	1.41	9.99	±	18.15	1.10	1.35	±	2.85	0.57	37	37%
4	**Fish and fish products**	**16.66**	±	**5.06**	**2.45**	±	**5.55**	**0.27**	**0.57**	±	**1.38**	**0.24**	**25**	25%
5	**Fruits and fruit products**	**23.53**	±	**26.71**	**116.41**	±	**116.29**	**12.81**	**14.76**	±	**22.04**	**6.26**	**99**	99%
	composite food, mix fruit	9.33	±	0.00	1.00	±	7.67	0.11	0.33	±	2.83	0.14	5	5%
	dishes with peanut sauce, fruit, mango	0.00	±	0.00	0.59	±	4.76	0.06	0.37	±	3.24	0.16	2	2%
	dishes with peanut sauce, mix	59.17	±	0.00	1.73	±	5.04	0.19	1.08	±	3.66	0.46	18	18%

No	Food Groups	PS content (mg/100g of food)			Food Consumption (g/person/day)			% Total Consumption	PS Intake (mg/person/day)			% Total Intake	No. of Eater (N=100)	% of Eater (N=100)
		Mean	±	S.D.	Mean	±	S.D.		Mean	±	S.D.			
	fruits													
	fruits, processed, dried	62.00	±	0.00	0.13	±	0.85	0.01	0.08	±	0.53	0.03	4	4%
	fruits, raw	20.90	±	27.08	112.75	±	113.13	12.41	12.88	±	19.00	5.46	99	99%
	jam and jelly	12.00	±	0.00	0.22	±	1.44	0.02	0.03	±	0.17	0.01	6	6%
**6**	**Herbs, spices and condiments**	**44.08**	±	**66.76**	**13.88**	±	**18.59**	**1.53**	**6.39**	±	**8.86**	**2.71**	**79**	79%
	condiments	63.66	±	72.68	9.45	±	11.14	1.04	6.37	±	8.82	2.70	79	79%
	herbs and spices	0.00	±	0.00	0.06	±	0.38	0.01	0.00	±	0.00	0.00	3	3%
	traditional herbal drinks	0.00	±	0.00	4.37	±	13.66	0.48	0.02	±	0.10	0.01	23	23%
**7**	**Legumes and legume products**	**89.40**	±	**115.35**	**84.66**	±	**72.77**	**9.32**	**55.62**	±	**52.06**	**23.59**	**98**	98%
	composite food, oncom based	0.85	±	1.21	4.20	±	7.82	0.46	0.44	±	0.86	0.19	42	42%
	composite food, red bean soup	127.00	±	0.00	0.33	±	2.35	0.04	0.12	±	0.87	0.05	2	2%
	composite food, tempeh based	95.63	±	53.89	31.69	±	37.13	3.49	41.31	±	49.57	17.52	79	79%
	composite food, tofu based	8.51	±	5.02	21.36	±	15.80	2.35	2.77	±	2.84	1.17	86	86%
	legumes, mung bean based	11.16	±	6.02	16.81	±	39.06	1.85	1.93	±	4.43	0.82	44	44%
	legumes, other (cashew, fava, etc.)	195.20	±	196.50	0.76	±	2.15	0.08	1.04	±	2.85	0.44	17	17%
	legumes, peanut based	141.25	±	83.34	3.44	±	6.65	0.38	7.39	±	14.40	3.14	39	39%
	legumes, soy based	5.56	±	0.00	6.08	±	28.27	0.67	0.61	±	2.45	0.26	14	14%
**8**	**Meat and meat products**	**20.98**	±	**22.13**	**33.65**	±	**36.58**	**3.70**	**4.42**	±	**4.89**	**1.87**	**86**	86%
	composite food, meat based	7.90	±	3.74	16.75	±	33.08	1.84	2.03	±	4.47	0.86	35	35%
	composite food, poultry based	9.42	±	2.14	4.43	±	9.83	0.49	0.48	±	1.06	0.21	33	33%
	dishes with peanut sauce, meat or poultry based	62.37	±	0.98	0.81	±	2.61	0.09	0.22	±	0.68	0.10	16	16%
	fried dishes, poultry based	14.17	±	3.72	11.67	±	12.09	1.29	1.68	±	1.76	0.71	68	68%
**9**	**Phytosterol fortified products**	**88.90**	±	**0.00**	**0.03**	±	**0.30**	**0.00**	**0.03**	±	**0.27**	**0.01**	**1**	1%
**10**	**Snack foods**	**29.88**	±	**35.01**	**106.91**	±	**99.39**	**11.77**	**36.35**	±	**34.70**	**15.42**	**99**	99%
	biscuits	30.00	±	0.00	5.51	±	12.65	0.61	1.65	±	3.79	0.70	39	39%
	bread	0.00	±	0.00	13.19	±	23.44	1.45	0.24	±	0.66	0.10	52	52%

No	Food Groups	PS content (mg/100g of food)			Food Consumption (g/person/day)			% Total Consumption	PS Intake (mg/person/day)			% Total Intake	No. of Eater (N=100)	% of Eater (N=100)
		Mean	±	S.D.	Mean	±	S.D.		Mean	±	S.D.			
	dessert	0.00	±	0.00	0.18	±	1.76	0.02	0.00	±	0.00	0.00	1	1%
	fried snacks, plantain based	23.69	±	0.00	2.75	±	9.23	0.30	0.75	±	2.53	0.32	12	12%
	fried snacks, rice flour based	25.43	±	16.10	1.13	±	3.17	0.12	0.25	±	0.89	0.11	19	19%
	fried snacks, soy based	53.60	±	58.96	28.69	±	40.17	3.16	21.34	±	29.24	9.05	73	73%
	fried snacks, tuber based	18.07	±	13.00	14.14	±	21.43	1.56	1.97	±	3.13	0.83	66	66%
	fried snacks, wheat flour based	52.28	±	48.83	33.42	±	48.29	3.68	6.66	±	8.88	2.82	82	82%
	other bakery wares	30.56	±	27.76	6.21	±	12.22	0.68	2.90	±	5.29	1.23	64	64%
	sweets	73.33	±	24.83	0.49	±	2.14	0.05	0.32	±	1.34	0.13	11	11%
	traditional cake and snacks	18.59	±	8.43	1.20	±	7.55	0.13	0.27	±	1.61	0.11	8	8%
**11**	**Supplements**	**0.00**	±	**0.00**	**0.12**	±	**0.45**	**0.01**	**0.00**	±	**0.00**	**0.00**	**9**	9%
**12**	**Vegetables and vegetable products**	**22.10**	±	**26.72**	**142.57**	±	**98.44**	**15.69**	**28.48**	±	**24.76**	**12.08**	**100**	100%
	composite food, mix vegetables soup	23.86	±	14.67	28.17	±	23.20	3.10	5.81	±	5.52	2.46	93	93%
	composite food, mix vegetables, raw or steamed	22.92	±	21.33	0.82	±	5.81	0.09	0.30	±	2.24	0.13	4	4%
	composite food, mix vegetables, stir fried	10.83	±	0.00	2.91	±	5.75	0.32	0.46	±	0.91	0.19	38	38%
	dishes with peanut sauce, leafy or stem vegetable	43.72	±	-	0.18	±	1.14	0.02	0.08	±	0.50	0.03	3	3%
	dishes with peanut sauce, mix vegetable	45.70	±	13.68	29.15	±	45.97	3.21	11.39	±	18.33	4.83	70	70%
	vegetables, processed, fermented	0.00	±	-	0.03	±	0.19	0.00	0.00	±	0.00	0.00	2	2%
	vegetables, processed, fried	23.77	±	19.07	14.28	±	26.40	1.57	2.19	±	8.79	0.93	67	67%
	vegetables, processed, steamed	12.00	±	-	5.02	±	11.26	0.55	0.60	±	1.35	0.26	29	29%
	vegetables, processed, boiled	8.99	±	6.68	21.15	±	25.44	2.33	1.03	±	1.51	0.44	77	77%
	vegetables, processed, stir fried	27.23	±	40.69	21.38	±	18.31	2.35	4.02	±	3.73	1.70	89	89%
	vegetables, raw	24.97	±	28.97	19.49	±	33.72	2.15	2.61	±	4.01	1.11	72	72%
	**TOTAL**	**32.64**	±	**53.05**	**908.43**	±	**349.83**	**100.00**	**235.73**	±	**104.52**	**100.00**	**100**	**100%**

Table S6. Plant sterols (PS) content in every food item consumed by the respondents.

No	Group	Sub-Group	Food Name	Data Source*	PS content (mg/100g of food)	Consumed in Rural Area	Consumed in Urban Area
1	beverages	-	coffee, black	h	0.00	√	√
2	beverages	-	coffee, with milk	h	0.00	√	√
3	beverages	-	ENERGEN, instant cereal drink, chocolate or other flavor	a	0.00	√	√
4	beverages	-	milk and ginger drink	h	0.00	X	√
5	beverages	-	milk, chocolate, powder	h	0.00	√	X
6	beverages	-	milk, full cream, powder	h	0.00	√	√
7	beverages	-	milk, liquid	a	0.00	√	√
8	beverages	-	soft drinks	h	0.00	√	X
9	beverages	-	sweet condensed milk	h	0.00	√	√
10	beverages	-	syrup	h	0.00	√	√
11	beverages	-	syrup ice, served with condensed milk, black rice, and jackfruit (es doger)	e	2.49	√	X
12	beverages	-	tea, plain	h	0.00	√	√
13	beverages	-	tea, prepared with sugar	h	0.00	√	√
14	Cereals and cereal products	cereal product, brown rice based	rice, brown, boiled	b	26.80	√	√
15	Cereals and cereal products	cereal product, corn based	corn, dried, fried with seasoning	e	50.67	X	√
16	Cereals and cereal products	cereal product, corn based	corn, grilled	c**	28.50	√	√
17	Cereals and cereal products	cereal product, corn based	corn, steamed	c**	28.50	√	√
18	Cereals and cereal products	cereal product, corn based	corn, steamed, served with grated coconut	e	22.13	√	√
19	Cereals and cereal products	cereal product, oat based	oatmeal	b	44.00	√	√

No	Group	Sub-Group	Food Name	Data Source*	PS content (mg/100g of food)	Consumed in Rural Area	Consumed in Urban Area
20	Cereals and cereal products	cereal product, processed	instant, noodle, drained	e	20.61	√	√
21	Cereals and cereal products	cereal product, processed	instant, noodle, soup	e	20.61	√	√
22	Cereals and cereal products	cereal product, rice based	rice, black, steamed	b	26.80	√	√
23	Cereals and cereal products	cereal product, rice based	rice, sticky, fried	e	43.71	√	√
24	Cereals and cereal products	cereal product, rice based	rice, sticky, steamed, served with grated coconut	e	35.80	√	√
25	Cereals and cereal products	cereal product, rice based	rice, white, boiled	b	22.50	√	√
26	Cereals and cereal products	cereal product, rice flour based	rice noodle, with vegetable and soy sauce, boiled	a	22.50	√	√
27	Cereals and cereal products	cereal product, rice flour based	rice noodle, with vegetable and soy sauce, fried	e	26.21	√	√
28	Cereals and cereal products	cereal product, wheat flour based	macaroni, fried	e	44.83	X	√
29	Cereals and cereal products	cereal product, wheat flour based	pasta	b	28.10	√	X
30	Cereals and cereal products	cereal product, wheat flour based	pasta with sauce	e	46.86	X	√
31	Cereals and cereal products	cereal product, wheat flour based	wheat flour based, chocolate breakfast cereal	a	0.00	√	X
32	Cereals and cereal products	composite food, rice based	chicken rice porridge	e	120.19	√	√
33	Cereals and cereal products	composite food, rice based	fried rice	e	20.17	√	X
34	Cereals and cereal products	composite food, rice based	rice cake with oncom filling	e	26.89	√	√

No	Group	Sub-Group	Food Name	Data Source*	PS content (mg/100g of food)	Consumed in Rural Area	Consumed in Urban Area
35	cereals and cereal products	composite food, rice based	Rice cake with vegetable and coconut milk soup	e	35.51	√	√
36	cereals and cereal products	composite food, rice based	rice, in coconut milk, served with side dishes	e	16.83	√	X
37	cereals and cereal products	composite food, rice flour based	river rice noodles, with vegetable and soy sauce, fried	e	31.81	√	√
38	cereals and cereal products	composite food, wheat flour based	chicken noodle	e	19.07	X	√
39	cereals and cereal products	composite food, wheat flour based	noodle, with vegetable and soy sauce, fried	e	25.45	√	√
40	cereals and cereal products	composite food, wheat flour based	noodle, with vegetable soup	e	14.98	√	√
41	cereals and cereal products	composite food, wheat flour based	wheat flour sheet, filled with vegetables	e	15.89	X	√
42	cereals and cereal products	dishes with peanut sauce, rice based	rice cake, rice noodles, and tofu, steamed, served with peanut sauce and vegetables	e	37.91	√	√
43	cereals and cereal products	dishes with peanut sauce, rice based	rice cake, served with peanut sauce	e	44.91	√	√
44	cereals and cereal products	dishes with peanut sauce, wheat flour based	wheat flour snacks, dim sum, served with peanut sauce	e	50.56	√	√
45	eggs and egg products	composite food, egg	egg in chili sauce	e	8.02	X	√
46	eggs and egg products	egg, boiled	egg, boiled	h	0.00	X	√
47	eggs and egg products	fried dishes, egg	Egg, whole, cooked, fried	a	8.00	√	√
48	eggs and egg products	fried dishes, egg	Egg, whole, cooked, omelet	a	10.00	√	√
49	fish and fish products	-	bloat fish, fried	e	18.33	X	√
50	fish and fish products	-	carp fish, prepared with spices, fried	e	18.33	√	√
51	fish and fish products	-	fish, salted, fried	e	6.45	√	√
52	fish and fish products	-	prawn, fried	e	18.33	X	√

No	Group	Sub-Group	Food Name	Data Source*	PS content (mg/100g of food)	Consumed in Rural Area	Consumed in Urban Area
53	fish and fish products	-	tilapia fish, prepared with spices, fried	e	18.33	√	√
54	fish and fish products	-	tuna fish, salted, fried	e	20.20	√	√
55	fruits and fruit products	composite food, mix fruit	mix all fruits, served sour soup	e	9.33	√	√
56	fruits and fruit products	composite food, mix fruit	mix all fruits, served with ice	e	22.64	√	X
57	fruits and fruit products	dishes with peanut sauce, fruit	mango, served with salt and peanut sauce	n.a	n.a	X	√
58	fruits and fruit products	dishes with peanut sauce, mix fruits	mix all fruits, served with peanut sauce (Rujak)	e	59.17	√	√
59	fruits and fruit products	fruits, processed, dried	candied fruit	a	62.00	√	√
60	fruits and fruit products	fruits, raw	apples, raw	a	12.00	√	√
61	fruits and fruit products	fruits, raw	avocados, raw	a	83.00	√	√
62	fruits and fruit products	fruits, raw	avocados, raw, with sugar	a	83.00	√	√
63	fruits and fruit products	fruits, raw	bananas, raw	a	16.00	√	√
64	fruits and fruit products	fruits, raw	bengkuang, yambeans, raw	a	10.00	√	X
65	fruits and fruit products	fruits, raw	carambolas, raw	n.a	n.a	X	√
66	fruits and fruit products	fruits, raw	coconuts, flesh	a	47.00	√	X
67	fruits and fruit products	fruits, raw	custard apple, soursops, raw	n.a	n.a	√	√
68	fruits and fruit products	fruits, raw	custard apple, soursops, raw, with sugar	n.a	n.a	√	√
69	fruits and fruit products	fruits, raw	durian	n.a	n.a	√	√
70	fruits and fruit products	fruits, raw	grapes, raw	a	4.00	√	√
71	fruits and fruit products	fruits, raw	guavas, raw	n.a	n.a	√	√
72	fruits and fruit products	fruits, raw	guavas, raw, with sugar	n.a	n.a	√	X
73	fruits and fruit products	fruits, raw	jackfruits, raw	n.a	n.a	√	√
74	fruits and fruit products	fruits, raw	lime	c	22.80	X	√
75	fruits and fruit products	fruits, raw	longans, raw	n.a	n.a	√	√

No	Group	Sub-Group	Food Name	Data Source*	PS content (mg/100g of food)	Consumed in Rural Area	Consumed in Urban Area
76	fruits and fruit products	fruits, raw	mangos, raw	n.a	n.a	√	√
77	fruits and fruit products	fruits, raw	melons, raw	a	10.00	√	√
78	fruits and fruit products	fruits, raw	oranges, raw	c**	22.80	√	√
79	fruits and fruit products	fruits, raw	papayas, raw	n.a	n.a	√	√
80	fruits and fruit products	fruits, raw	pears, raw	a	8.00	√	√
81	fruits and fruit products	fruits, raw	persimmons, raw	a	4.00	√	√
82	fruits and fruit products	fruits, raw	pineapples, raw	a	6.00	√	X
83	fruits and fruit products	fruits, raw	pineapples, raw	a	6.00	X	√
84	fruits and fruit products	fruits, raw	rambutans, raw	n.a	n.a	√	√
85	fruits and fruit products	fruits, raw	rose apples, raw	n.a	n.a	√	X
86	fruits and fruit products	fruits, raw	sapodillas, raw	n.a	n.a	√	X
87	fruits and fruit products	fruits, raw	snakeskin, salak fruits, raw	n.a	n.a	√	√
88	fruits and fruit products	fruits, raw	strawberries, raw	a	12.00	√	√
89	fruits and fruit products	fruits, raw	strawberries, raw, with sugar	c**	10.00	√	X
90	fruits and fruit products	fruits, raw	tomatoes, sliced, prepared with sugar	a	7.00	√	√
91	fruits and fruit products	fruits, raw	watermelons, raw	a	2.00	√	√
92	fruits and fruit products	jam and jelly	pineapple jam	a	12.00	X	√
93	fruits and fruit products	jam and jelly	strawberry jam	a	12.00	X	√
94	herbs, spices and condiments	condiments	chili and nut paste, prepared with spices	e	200.11	√	√
95	herbs, spices and condiments	condiments	chili and soy sauce, prepared with spices, for dipping	e	29.88	√	X
96	herbs, spices and condiments	condiments	chili paste, prepared with spices, stir fried	e	33.87	√	√
97	herbs, spices and condiments	condiments	chili sauce	a	7.00	√	√
98	herbs, spices and condiments	condiments	margarine	a	146.00	√	√

No	Group	Sub-Group	Food Name	Data Source*	PS content (mg/100g of food)	Consumed in Rural Area	Consumed in Urban Area
**99**	herbs, spices and condiments	condiments	mayonnaise	a	97.00	X	√
**100**	herbs, spices and condiments	condiments	soy sauce	h	0.00	√	√
**101**	herbs, spices and condiments	condiments	soybean paste	n.a	n.a	√	√
**102**	herbs, spice, and condiments	condiments	tomato sauce	a	7.00	√	√
**103**	herbs, spices and condiments	condiments	turmeric, raw	a	82.00	√	√
**104**	herbs, spices and condiments	condiments	vinegar	a	0.00	√	√
**105**	herbs, spices and condiments	herbs and spices	betel leaf	n.a	n.a	X	√
**106**	herbs, spices and condiments	herbs and spices	lempuyang, Zingiber zerumbet L	n.a	n.a	X	√
**107**	herbs, spices and condiments	herbs and spices	sambiloto, Andrographis paniculata	n.a	n.a	X	√
**108**	herbs, spices and condiments	traditional herbal drinks	galangal, extract, drinking liquid	h	0.00	√	√
**109**	herbs, spices and condiments	traditional herbal drinks	ginger, extract, drinking liquid	h	0.00	√	√
**110**	herbs, spices and condiments	traditional herbal drinks	Indonesian hot spiced drink	h	0.00	√	X
**111**	herbs, spices and condiments	traditional herbal drinks	mix herbs, extract, drinking liquid	h	0.00	√	√
**112**	herbs, spices and condiments	traditional herbal drinks	sour and turmeric, extract, drinking liquid	h	0.00	√	√
**113**	legumes and legume products	composite food, oncom based	oncom based, stir fried	e	1.71	√	√

No	Group	Sub-Group	Food Name	Data Source*	PS content (mg/100g of food)	Consumed in Rural Area	Consumed in Urban Area
114	legumes and legume products	composite food, oncom based	oncom, steamed and spiced	h	0.00	√	√
115	legumes and legume products	composite food, peanut based	peanut, with salted fish, fried	e	131.76	√	X
116	legumes and legume products	composite food, red bean soup	Kidney bean soup	a	127.00	√	√
117	legumes and legume products	composite food, tempeh based	tempeh in chili sauce	e	87.05	√	√
118	legumes and legume products	composite food, tempeh based	tempeh, fried	e	148.23	√	√
119	legumes and legume products	composite food, tempeh based	tempeh, in soy sauce soup, fried	e	123.14	√	√
120	legumes and legume products	composite food, tempeh based	tempeh, with soy sauce soup	e	135.18	√	X
121	legumes and legume products	composite food, tempeh based	tempeh, with soy sauce, stir fried	e	24.10	√	√
122	legumes and legume products	composite food, tofu based	fried tofu, served with spice and sour sauce	e	45.64	√	X
123	legumes and legume products	composite food, tofu based	tofu in red pepper sauce	e	14.47	√	√
124	legumes and legume products	composite food, tofu based	tofu, fried	e	9.49	√	√
125	legumes and legume products	composite food, tofu based	tofu, in soy sauce soup, fried	e	11.11	√	√
126	legumes and legume products	composite food, tofu based	tofu, steamed and spiced, wrapped in banana leaf	e	10.89	√	√
127	legumes and legume products	composite food, tofu based	tofu, stir fried	e	0.82	√	√
128	legumes and legume products	composite food, tofu based	tofu, with soy sauce soup	e	4.28	√	√

No	Group	Sub-Group	Food Name	Data Source*	PS content (mg/100g of food)	Consumed in Rural Area	Consumed in Urban Area
129	legumes and legume products	legumes, mung bean based	mung bean milk	a	6.90	√	√
130	legumes and legume products	legumes, mung bean based	mung bean porridge	e	15.41	√	√
131	legumes and legume products	legumes, other	bean, kidney, boiled	a	127.00	√	√
132	legumes and legume products	legumes, other	bean, pea, fried	a	135.00	√	√
133	legumes and legume products	legumes, other	cashew, roasted	a	158.00	√	√
134	legumes and legume products	legumes, other	sunflower seed	a	534.00	√	√
135	legumes and legume products	legumes, other	Beans, fava, boiled	a	22.00	X	√
136	legumes and legume products	legumes, peanut based	peanut butter	a	102.00	√	√
137	legumes and legume products	legumes, peanut based	peanut, fried	e	205.52	√	√
138	legumes and legume products	legumes, peanut based	peanut, steamed	a	220.00	√	√
139	legumes and legume products	legumes, peanut based	peanut, covered with flour, fried	a	220.00	X	√
140	legumes and legume products	legumes, peanut based	soy bean	a	50.00	√	√
141	legumes and legume products	legumes, soy based	soy milk	e	5.56	√	√
142	meat and meat products	composite food, meat based	meatball with noodle and vegetable in soup	e	11.07	√	√
143	meat and meat products	composite food, meat based	spiced meat/meat offal soup	e	3.77	√	√

No	Group	Sub-Group	Food Name	Data Source*	PS content (mg/100g of food)	Consumed in Rural Area	Consumed in Urban Area
144	meat and meat products	composite food, meat based	spiced noodle soup	e	8.87	√	√
145	meat and meat products	composite food, poultry based	spiced chicken/chicken offal soup	e	10.93	√	√
146	meat and meat products	composite food, poultry based	spiced chicken/chicken offal with coconut milk soup	e	7.91	√	√
147	meat and meat products	dishes with peanut sauce, meat or poultry based	chicken satai, grilled, served with peanut sauce	e	63.07	√	√
148	meat and meat products	dishes with peanut sauce, meat or poultry based	lamb satai, grilled, served with peanut sauce	e	61.68	√	√
149	meat and meat products	fried dishes, poultry based	chicken, prepared with soy sauce and spices, fried	e	9.95	X	√
150	meat and meat products	fried dishes, poultry based	chicken, prepared with spices and flour, fried	e	16.97	X	√
151	meat and meat products	fried dishes, poultry based	chicken, prepared with spices, fried	e	15.58	X	√
152	meat and meat products	composite food, meat based	spiced meat/meat offal with coconut milk soup	e	4.63	√	X
153	meat and meat products	composite food, poultry based	chicken, boiled, with coconut milk and spices soup	e	2.57	√	X
154	meat and meat products	fried dishes, meat based	meat, prepared with spices, fried, steamed	e	5.55	√	X
155	meat and meat products	fried dishes, poultry based	chicken, prepared with spices and flour, fried, steamed	e	16.97	√	X
156	meat and meat products	fried dishes, poultry based	chicken, prepared with spices, fried, steamed	e	15.58	√	X
157	phytosterol fortified products	-	Tropicana slim non-fat fitosterol	f	88.90	√	√

No	Group	Sub-Group	Food Name	Data Source*	PS content (mg/100g of food)	Consumed in Rural Area	Consumed in Urban Area
158	snack foods	biscuits	biscuits, common	a	30.00	√	√
159	snack foods	biscuits	biscuits, with cheese cream	a	30.00	√	X
160	snack foods	biscuits	biscuits, with fruit jam	a	30.00	√	X
161	snack foods	biscuits	biscuits, with peanut butter	a	49.00	√	X
162	snack foods	biscuits	cracker	a	30.00	√	X
163	snack foods	bread	bread, beef floss filling	a	0.00	√	X
164	snack foods	bread	bread, cheese cream filling	a	0.00	√	√
165	snack foods	bread	bread, chocolate filling	a	0.00	√	√
166	snack foods	bread	bread, fruit jam filling	a	0.00	√	√
167	snack foods	bread	bread, margarine and chocolate rice filling	a	0.00	√	√
168	snack foods	bread	bread, mung bean filling	a	0.00	√	√
169	snack foods	bread	palm and cinnamon bread (roti gambang)	a	0.00	X	√
170	snack foods	bread	plain bread	a	0.00	X	√
171	snack foods	dessert	cheese sauce	a	11.00	√	X
172	snack foods	dessert	pudding	a	0.00	√	√
173	snack foods	fried snacks, plantain based	chips, plantain, fried	e	21.78	√	X
174	snack foods	fried snacks, plantain based	plantain, wheat flour, fried	e	23.69	√	√
175	snack foods	fried snacks, rice flour based	rice flour, fried	e	6.87	√	√
176	snack foods	fried snacks, rice flour based	rice, fried	e	59.16	√	X
177	snack foods	fried snacks, rice flour based	snacks, flour based, deep fried	e	35.63	√	√
178	snack foods	fried snacks, rice flour based	traditional cake, rice flour with palm sugar, fried	e	33.79	X	√
179	snack foods	fried snacks, rice flour	traditional cake, rice flour with palm sugar, fried (kue cucur)	e	33.79	√	X

No	Group	Sub-Group	Food Name	Data Source*	PS content (mg/100g of food)	Consumed in Rural Area	Consumed in Urban Area
		based					
180	snack foods	fried snacks, soy based	oncom based, with wheat flour, fried	e	100.23	√	√
181	snack foods	fried snacks, soy based	tempeh based, wheat flour, fried	e	133.53	√	√
182	snack foods	fried snacks, soy based	tofu based, wheat flour, filled with sprout and vegetables, fried	e	12.98	√	√
183	snack foods	fried snacks, soy based	tofu based, wheat flour, fried	e	11.54	√	√
184	snack foods	fried snacks, soy based	tofu dough, fried	e	9.74	X	√
185	snack foods	fried snacks, tuber based	cassava cake with oncom and spices, fried	e	6.87	√	√
186	snack foods	fried snacks, tuber based	cassava cake, fried	e	6.21	√	X
187	snack foods	fried snacks, tuber based	cassava, fried	e	38.95	√	√
188	snack foods	fried snacks, tuber based	chips, cassava, fried	a	12.00	√	√
189	snack foods	fried snacks, tuber based	chips, potato, fried	a	34.00	√	√
190	snack foods	fried snacks, tuber based	chips, sweet potato, fried	a	12.00	√	X
191	snack foods	fried snacks, tuber based	chips, taro, fried	e	20.64	√	√
192	snack foods	fried snacks, tuber based	French fries	a	0.00	√	√
193	snack foods	fried snacks, tuber based	sweet potato cake, fried	e	18.01	√	X
194	snack foods	fried snacks, tuber based	sweet potato, fried	e	18.85	√	√
195	snack foods	fried snacks, tuber based	taro, fried	e	24.43	√	√

No	Group	Sub-Group	Food Name	Data Source*	PS content (mg/100g of food)	Consumed in Rural Area	Consumed in Urban Area
196	snack foods	fried snacks, tuber based	traditional snack, cassava cake with palm sugar, fried	e	6.87	√	√
197	snack foods	fried snacks, wheat flour based	bread, with chocolate or fruit jam filling, fried	e	10.70	√	X
198	snack foods	fried snacks, wheat flour based	doughnut, fried, served with margarine and chocolate rice topping	e	48.36	√	X
199	snack foods	fried snacks, wheat flour based	fried mix vegetable, with wheat flour, fried	e	17.39	√	√
200	snack foods	fried snacks, wheat flour based	snacks, flour based, prepared with peanut, deep fried	e	131.74	√	√
201	snack foods	fried snacks, wheat flour based	snacks, flour based, prepared with salted fish, deep fried	e	18.97	√	X
202	snack foods	fried snacks, wheat flour based	traditional snack, wheat flour, fried	e	53.07	√	X
203	snack foods	fried snacks, wheat flour based	wheat flour based, banana and chocolate rice, fried	e	31.64	√	X
204	snack foods	fried snacks, wheat flour based	wheat flour based, egg and vegetables, fried	e	37.02	√	√
205	snack foods	fried snacks, wheat flour based	wheat flour based, filled with rice noodle and vegetables, fried	e	10.09	√	√
206	snack foods	fried snacks, wheat flour based	wheat flour based, filled with vegetables or egg, fried	e	24.28	√	√
207	snack foods	fried snacks, wheat flour based	wheat flour based, sweet corn, fried	e	33.09	√	√
208	snack foods	fried snacks, wheat flour based	wheat flour dough, semi pastry, fried	e	112.37	X	√
209	snack foods	other bakery wares	cake, common	b	28.00	√	√
210	snack foods	other bakery wares	cookies	a	0.00	√	√
211	snack foods	other bakery wares	pancake, plain	e	1.58	√	√

No	Group	Sub-Group	Food Name	Data Source*	PS content (mg/100g of food)	Consumed in Rural Area	Consumed in Urban Area
212	snack foods	other bakery wares	pancake, with cheese and condensed milk	e	42.65	√	√
213	snack foods	other bakery wares	pancake, with peanut, chocolate rice, and sugar	e	73.96	√	√
214	snack foods	other bakery wares	pancake, with sticky rice and condensed milk	e	66.75	√	X
215	snack foods	other bakery wares	wafers, chocolate	b	37.20	√	√
216	snack foods	sweets	chocolate rice	a	59.00	√	√
217	snack foods	sweets	dark chocolate	a	102.00	X	√
218	snack foods	sweets	milk chocolate	a	59.00	√	√
219	snack foods	traditional cake and snacks	traditional cake, cassava based, steamed (Ketimus)	e	0.00	√	X
220	snack foods	traditional cake and snacks	traditional cake, cassava, mashed, steamed (Getuk)	n.a	n.a	√	X
221	snack foods	traditional cake and snacks	traditional cake, pie, mung bean/chocolate/black bean filling (Kue Pia)	e	18.80	√	X
222	snack foods	traditional cake and snacks	traditional cake, rice flour and grated coconut, pan fried (Kue Pancong)	e	31.21	√	√
223	snack foods	traditional cake and snacks	traditional cake, rice flour based, steamed (Kue Lopis)	e	31.32	√	X
224	snack foods	traditional cake and snacks	traditional cake, rice flour, steamed (Kue Apem)	e	14.80	√	√
225	snack foods	traditional cake and snacks	traditional cake, rice flour, filled with grated coconut and palm sugar (Kue Bugis)	e	14.80	X	√
226	snack foods	traditional cake and snacks	traditional cake, spekkoek, layer cake (Lapis Legit)	e	68.45	√	X
227	snack foods	traditional cake and snacks	traditional cake, wheat flour and banana, steamed (Nagasari)	e	8.00	√	X
228	snack foods	traditional cake and snacks	traditional cake, wheat flour pan fried (Kue Ape)	e	13.55	√	√
229	supplements	-	chlorophyll drink	h	0.00	√	X
230	supplements	-	enervon-C	h	0.00	√	X

No	Group	Sub-Group	Food Name	Data Source*	PS content (mg/100g of food)	Consumed in Rural Area	Consumed in Urban Area
231	supplements	Herbal supplement	Habbatussauda	h	0.00	X	√
232	supplements	Iron tablet	Iron tablet	h	0.00	X	√
233	supplements	Iron tablet	SANGOBION	h	0.00	X	√
234	supplements	multivitamin	HEMAVITON	h	0.00	X	√
235	supplements	multivitamin	multivitamin	h	0.00	X	√
236	supplements	vitamin C	vitamin C	h	0.00	X	√
237	vegetables and vegetable products	composite food, mix vegetables soup	mix all vegetables soup, soup only	e	6.61	√	X
238	vegetables and vegetable products	composite food, mix vegetables soup	mix all vegetables, served sour soup	e	28.72	√	√
239	vegetables and vegetable products	composite food, mix vegetables soup	mix all vegetables, served with vinegar and onion	e	19.75	√	√
240	vegetables and vegetable products	composite food, mix vegetables soup	mix all vegetables, soup	e	12.83	√	√
241	vegetables and vegetable products	composite food, mix vegetables soup	mix vegetables in coconut milk soup	e	44.83	√	√
242	vegetables and vegetable products	composite food, mix vegetables soup	mix vegetables soup, with chicken/meat/meatball added	e	9.10	√	√
243	vegetables and vegetable products	composite food, mix vegetables soup	Mixed vegetables sour soup	e	41.12	√	√
244	vegetables and vegetable products	composite food, mix vegetables soup	rice noodle and sprout spicy soup, with or without chicken/prawn/meat/egg added (laksa)	e	10.68	√	√
245	vegetables and vegetable products	composite food, mix vegetables, raw or steamed	mix all vegetables, steamed, served with grated coconut	e	7.83	√	√
246	vegetables and vegetable products	composite food, mix vegetables, raw or steamed	vegetable salad	e	38.00	X	√
247	vegetables and vegetable	composite food, mix	mix all vegetables, stir fried	e	10.83	√	√

No	Group	Sub-Group	Food Name	Data Source*	PS content (mg/100g of food)	Consumed in Rural Area	Consumed in Urban Area
	products	vegetables, stir fried					
248	vegetables and vegetable products	dishes with peanut sauce, leafy or stem vegetable	sprout, stir fried, with peanut sauce	e	43.72	X	√
249	vegetables and vegetable products	dishes with peanut sauce, mix vegetable	mix all vegetables, served with peanut sauce (Gado-gado)	e	34.80	√	√
250	vegetables and vegetable products	dishes with peanut sauce, mix vegetable	mix all vegetables, served with peanut sauce (Karedok)	e	41.25	√	√
251	vegetables and vegetable products	dishes with peanut sauce, mix vegetable	mix all vegetables, served with peanut sauce (Pecel)	e	61.04	√	√
252	vegetables and vegetable products	vegetables, processed, fermented	cassava, fermented	h	0.00	√	√
253	vegetables and vegetable products	vegetables, processed, fried	mushroom, deep fried	e	36.57	X	√
254	vegetables and vegetable products	vegetables, processed, fried	potato in chili sauce, with or without chicken oval	e	5.49	√	√
255	vegetables and vegetable products	vegetables, processed, fried	potato, mashed, battered with egg, fried	e	9.51	√	√
256	vegetables and vegetable products	vegetables, processed, fried	spinach, battered with flour, deep fried	e	43.50	X	√
257	vegetables and vegetable products	vegetables, processed, fried	sweet potato, mashed, fried	e	28.22	√	X
258	vegetables and vegetable products	vegetables, processed, steamed	sweet potato, steamed	a	12.00	√	√
259	vegetables and vegetable products	vegetables, processed, steamed or boiled	bamboo shoots, stir fried	e	10.82	√	√
260	vegetables and vegetable products	vegetables, processed, steamed or boiled	cassava leaves, with soup	e	0.44	√	√
261	vegetables and vegetable products	vegetables, processed, steamed or boiled	cassava, boiled, with soy sauce soup	e	8.15	√	√

No	Group	Sub-Group	Food Name	Data Source*	PS content (mg/100g of food)	Consumed in Rural Area	Consumed in Urban Area
262	vegetables and vegetable products	vegetables, processed, steamed or boiled	cassava, steamed	n.a	n.a	√	√
263	vegetables and vegetable products	vegetables, processed, steamed or boiled	cassava, steamed, mashed, served with grated coconut	e	3.36	√	√
264	vegetables and vegetable products	vegetables, processed, steamed or boiled	Chinese cabbage, boiled, with soup	e	11.41	√	√
265	vegetables and vegetable products	vegetables, processed, steamed or boiled	cucumbers, stir fried	e	21.96	√	√
266	vegetables and vegetable products	vegetables, processed, steamed or boiled	jackfruit, boiled, with coconut milk soup	e	2.15	√	√
267	vegetables and vegetable products	vegetables, processed, steamed or boiled	katuk leaf, soup	e	8.30	√	√
268	vegetables and vegetable products	vegetables, processed, steamed or boiled	mushroom, soup	e	2.52	√	√
269	vegetables and vegetable products	vegetables, processed, steamed or boiled	oyong, soup	e	18.16	√	√
270	vegetables and vegetable products	vegetables, processed, steamed or boiled	potato, steamed	a	5.00	√	√
271	vegetables and vegetable products	vegetables, processed, steamed or boiled	spinach, boiled, with soup, with/without corn added	e	8.20	√	√
272	vegetables and vegetable products	vegetables, processed, steamed or boiled	sweet potato, boiled with palm sugar and coconut milk	e	6.42	√	√
273	vegetables and vegetable products	vegetables, processed, steamed or boiled	taro, steamed	a	19.00	√	√
274	vegetables and vegetable products	vegetables, processed, steamed or boiled	taro, steamed, mashed, served with grated coconut	e	20.19	√	X
275	vegetables and vegetable products	vegetables, processed, stir fried	beans, broadbeans, green, stir fried	e	39.60	√	√
276	vegetables and vegetable products	vegetables, processed, stir fried	beans, broadbeans, steamed	a	35.00	√	√

No	Group	Sub-Group	Food Name	Data Source*	PS content (mg/100g of food)	Consumed in Rural Area	Consumed in Urban Area
277	vegetables and vegetable products	vegetables, processed, stir fried	beans, longbean, stir fried	e	27.15	√	√
278	vegetables and vegetable products	vegetables, processed, stir fried	bitter gourd, stir fried	e	1.74	√	X
279	vegetables and vegetable products	vegetables, processed, stir fried	brassica leaves, stir fried	e	23.35	√	√
280	vegetables and vegetable products	vegetables, processed, stir fried	broccoli, stir fried	e	24.46	√	√
281	vegetables and vegetable products	vegetables, processed, stir fried	cabbage, stir fried	e	11.26	√	√
282	vegetables and vegetable products	vegetables, processed, stir fried	carrot, stir fried	e	10.62	√	√
283	vegetables and vegetable products	vegetables, processed, stir fried	cauliflower, stir fried	e	12.71	√	X
284	vegetables and vegetable products	vegetables, processed, stir fried	chayote, steamed	a	12.00	√	√
285	vegetables and vegetable products	vegetables, processed, stir fried	chayote, stir fried	e	13.67	√	√
286	vegetables and vegetable products	vegetables, processed, stir fried	eggplant in chili sauce	e	15.62	√	√
287	vegetables and vegetable products	vegetables, processed, stir fried	eggplant in soy sauce soup	e	11.66	X	√
288	vegetables and vegetable products	vegetables, processed, stir fried	eggplants, stir fried	e	6.47	√	√
289	vegetables and vegetable products	vegetables, processed, stir fried	fern leaves, stir fried	e	1.80	√	√
290	vegetables and vegetable products	vegetables, processed, stir fried	paprika, stir fried	a	175.00	√	√
291	vegetables and vegetable products	vegetables, processed, stir fried	sprout, stir fried	e	11.08	√	√

No	Group	Sub-Group	Food Name	Data Source*	PS content (mg/100g of food)	Consumed in Rural Area	Consumed in Urban Area
292	vegetables and vegetable products	vegetables, processed, stir fried	water spinach, stir fried	e	17.01	√	√
293	vegetables and vegetable products	vegetables, processed, stir fried	yellow velvetleaf, stir fried	e	2.15	√	X
294	vegetables and vegetable products	vegetables, raw	allspice, raw	n.a	n.a	√	√
295	vegetables and vegetable products	vegetables, raw	bamboo shoots, raw	a	19.00	√	√
296	vegetables and vegetable products	vegetables, raw	basil leaves, raw	a	106.00	√	√
297	vegetables and vegetable products	vegetables, raw	broccoli, raw	c**	36.70	√	√
298	vegetables and vegetable products	vegetables, raw	cabbages, raw	a	11.00	√	√
299	vegetables and vegetable products	vegetables, raw	carrot, raw	i**	12.00	√	√
300	vegetables and vegetable products	vegetables, raw	cassava leaves, steamed	n.a	n.a	√	√
301	vegetables and vegetable products	vegetables, raw	Chinese cabbage, steamed	c**	13.00	√	X
302	vegetables and vegetable products	vegetables, raw	cucumbers, raw	a	14.00	√	√
303	vegetables and vegetable products	vegetables, raw	dogfruit, raw or stir fried	n.a	n.a	X	√
304	vegetables and vegetable products	vegetables, raw	eggplants, raw	a	7.00	√	√
305	vegetables and vegetable products	vegetables, raw	Lettuce, green leaf, raw	a	38.00	√	√
306	vegetables and vegetable products	vegetables, raw	papaya leaves, steamed	n.a	n.a	√	√

No	Group	Sub-Group	Food Name	Data Source*	PS content (mg/100g of food)	Consumed in Rural Area	Consumed in Urban Area
307	vegetables and vegetable products	vegetables, raw	sprout, raw or steamed	a	15.00	X	√
308	vegetables and vegetable products	vegetables, raw	tomatoes, raw	a	7.00	√	√
309	vegetables and vegetable products	vegetables, raw	water spinach, steamed	a	9.00	√	√

) note:** a = USDA SR-24; b = Normen *et al*. 2002; c = Piironen *et al*. 2003; d = http://cholesterol.about.com/od/dietitiansadvice/a/spices.htm; e = calculation based on recipe; f = commercial product available in the market; h = considered contained zero phytosterol; i = Piironen and Lampi 2004 *in* Dutta 2004; n.a = data not available; *) note:** Data had been processed.

Table S7. Example of plant sterols (PS) content calculation based on product or meal recipe.

Group	Sub-group	Food Item	Composition	PS content (mg/100 g)	% composi-tion	Estimated PS content (mg/100g of food item)
**Cereals and cereal products**	cereal product, corn based	corn, steamed, served with grated coconut	sweet corn	28.50	66.67	$\left(\frac{28.50}{100}x66.67\right)+0+(\frac{47.00}{100}x6.67)$ = 22.13
			sugar	0.00	26.67	
			coconut	47.00	6.67	
**Herbs, Spices and Condim ents**	Condi-ments	chili and nut paste, prepared with spices	peanuts	220.00	86.34	$\left(\frac{220}{100}x86.34\right)+\left(\frac{83}{100}*5.74\right)+(\frac{83}{100}*6.48)+(\frac{1}{100}x1.44)$ = 200.11
			cayenne pepper	83.00	5.74	
			red pepper	83.00	6.48	
			garlic	1.00	1.44	

Table S8. Plant sterols (PS) content based on calculation from the product or meal recipe.

No	Food Group	Sub-Group	Food Item	Composition	PS Content (mg/100 g)	% composition in food item	Estimated PS content (mg/100 g food item)
1	BEVERAGES						
		-	syrup ice, served with condensed milk, black rice, and jackfruit (es doger)	syrup	0	43.02	2.49
				sweetened condensed milk	0	9.3	
				syrup	0	5.81	
				water	0	23.26	
				fermented cassava	0	9.3	
				black rice	26.8	9.3	
2	CEREALS AND CEREAL PRODUCTS						
		cereal product, corn based	corn, steamed, served with grated coconut	sweet corn	28.5	66.67	22.13
				sugar	0	26.67	
				coconut	47	6.67	
		cereal product, rice based	rice, sticky, fried	glutinous rice	35.5	86.21	43.71
				cooking oil	95	13.79	
			rice, sticky, steamed, served with grated coconut	glutinous rice	35.5	97.4	35.8
				coconut	47	2.6	
		cereal product, rice flour based	rice noodle, with vegetable and soy sauce, fried	rice noodle	22.5	61.59	26.21
				seasoning	12.61	29.29	
				cooking oil	95	9.12	
		composite food, rice based	fried rice	eggs	0	22.48	20.17
				white rice	35.5	44.95	
				fried rice seasoning	11.03	31.84	
				cooking oil	95	0.74	
			rice cake with oncom filling	oncom	0	24.24	26.89
				white rice	35.5	75.76	
			Rice cake with vegetable and coconut milk soup	rice cake	35.5	60.49	35.51
				soup seasoning	15.85	14.76	
				chayote	12	13.75	
				condiment	38.82	2.29	
				crackers	35.63	6.42	
				fried onions	299.98	2.29	
			rice, in coconut milk, served with side dishes	rice	35.5	38.22	16.83
				seasoning	0.74	48.91	
				rice noodle	22.5	12.87	
			chicken rice porridge	rice porridge	17.75	62.96	120.19
				chicken porridge soup	9.04	38.3	
				fried onions	299.98	3.44	
				fried soybean	161	14.27	
				shredded chicken	0	53.24	
				celery	6	8.9	
				crackers	35.63	54.69	
				chili sauce	38.82	43.1	
				rice cake	35.5	100	
		composite food, rice flour based	river rice noodles, with vegetable and soy sauce, fried	kwetiaw	28.1	33.18	31.81
				cooking oil	95	14.93	
				cabbage	14.8	5.31	
				Chinese cabbage	13	6.97	
				salt	0	0.14	
				meatball	0	22.39	
				chili	83	7.96	
				eggs	0	9.12	
		composite food,	noodle, with vegetable	dried noodles	20.61	73.1	25.45

No	Food Group	Sub-Group	Food Item	Composition	PS Content (mg/100 g)	% composition in food item	Estimated PS content (mg/100 g food item)
		wheat flour based	and soy sauce, fried	leeks	19.4	4.39	
				cesim	13	7.31	
				cabbage	14.8	7.31	
				cooking oil	95	7.89	
			noodle, with vegetable soup	dried noodles	20.61	53.86	14.98
				eggs	0	29.62	
				pepper	92	2.69	
				sugar	0	2.69	
				salt	0	0.36	
				Chinese cabbage	13	10.77	
		dishes with peanut sauce, rice based	rice cake, rice noodles, and tofu, steamed, served with peanut sauce and vegetables	rice noodle	22.5	11.61	37.91
				rice cake	35.5	13.55	
				tofu	#N/A	13.55	
				bean sprouts	15	19.36	
				crackers	35.63	3.87	
				peanut sauce	68.84	38.06	
			rice cake, served with peanut sauce	rice cake	35.5	71.77	44.91
				peanut sauce	68.84	28.23	
		dishes with peanut sauce, wheat flour based	wheat flour snacks, dim sum, served with peanut sauce	what flour snacks, dim sum, fried	28.1	44.86	50.56
				peanut sauce	68.84	55.14	
3	FISH AND FISH PRODUCTS						
		-	carp fish, prepared with spices, fried	carp fish	3	83.33	18.33
				cooking oil	95	16.67	
			fish, salted, fried	salted fish	3	96.25	6.45
				cooking oil	95	3.75	
			tilapia fish, prepared with spices, fried	Tilapia	3	83.33	18.33
				cooking oil	95	16.67	
			tuna fish, salted, fried	tuna	3	81.3	20.2
				cooking oil	95	18.7	
4	FRUIT AND FRUIT PRODUCTS						
		composite food, mix fruit	mix all fruits, served sour soup	cucumber	14	25.37	9.33
				pineapple	6	23.79	
				yam beans	10	23.79	
				guava	#N/A	23.79	
				pickled soup	60.36	3.27	
			mix all fruits, served with ice	jackfruit	#N/A	15.5	22.64
				jelly powder	0	10.65	
				avocado	83	21.79	
				coconut	47	9.69	
				mango	#N/A	9.69	
				seaweed	#N/A	32.69	
		dishes with peanut sauce, mix fruits	mix all fruits, served with peanut sauce (Rujak)	cucumber	14	21.62	59.17
				guava	#N/A	21.62	
				mango	#N/A	10.81	
				yam beans	10	10.81	
				sweet potato	12	8.11	
				peanut sauce	200.11	27.03	
5	HERBS, SPICES AND CONDIMENTS						
		condiments	chili and nut paste, prepared with spices	peanuts	220	86.34	200.11

No	Food Group	Sub-Group	Food Item	Composition	PS Content (mg/100 g)	% composition in food item	Estimated PS content (mg/100 g food item)
				cayenne pepper	83	5.74	
				cayenne pepper	83	6.48	
				garlic	1	1.44	
			chili and soy sauce, prepared with spices	cayenne pepper	83	36	29.88
				soy sauce	0	64	
			chili paste, prepared with spices, stir fried	cayenne pepper	83	8.26	33.87
				red cayenne pepper	83	27.96	
				garlic	1	4.14	
				red onion	5	20.7	
				tomatoes	7	38.94	
6	MEATS AND MEAT PRODUCTS						
		composite food, meat based	meatball with noodle and vegetable in soup	Meatball	0	55.94	11.07
				fried onions	299.98	1.12	
				rice noodle	22.5	11.19	
				yellow noodles	20.61	11.19	
				mustard	13	8.39	
				celery	6	0.56	
				meatball soup	15.68	3.22	
				bean sprouts	15	8.39	
			spiced meat/meat offal soup	soto soup	11.67	19.76	3.77
				chicken oval	0	32.75	
				potato	5	10.92	
				tomatoes	7	8.73	
				celery	6	0.55	
				coconut milk	1	27.29	
			spiced meat/meat offal with coconut milk soup	spicy soup	11.67	18.26	4.63
				beef	0	30.27	
				gnetum gnemon cracker	35.63	2.52	
				celery	6	0.5	
				potato	5	15.14	
				tomatoes	7	8.07	
				coconut milk	1	25.23	
			spiced noodle soup	soto soup	11.67	19.23	8.87
				kikil	0	26.57	
				raw noodle	20.61	21.25	
				lumpia	7.66	15.94	
				tomatoes	7	7.97	
				potato	5	7.97	
				celery	6	1.06	
		composite food, poultry based	spiced chicken/chicken offal soup	spicy soup	11.67	31.7	10.93
				chicken	0	30.65	
				fried onions	299.98	1.75	
				potato	5	26.27	
				tomatoes	7	8.76	
				celery	6	0.88	
			spiced chicken/chicken offal with coconut milk soup	spicy soup	11.67	22.05	7.91
				coconut milk	1	30.45	
				chicken	0	21.32	
				fried onions	299.98	1.22	
				potato	5	18.27	
				tomatoes	7	6.09	
				celery	6	0.61	
			chicken, boiled, with coconut milk and spices soup	chicken	0	54.31	2.57
				seasoning	5.62	45.69	
		dishes with	lamb satai, grilled,	mutton	3	10.88	61.68

No	Food Group	Sub-Group	Food Item	Composition	PS Content (mg/100 g)	% composition in food item	Estimated PS content (mg/100 g food item)
		peanut sauce, meat or poultry based	served with peanut sauce	peanut sauce	68.84	89.12	
			chicken satai, grilled, served with peanut sauce	chicken	0	8.39	63.07
				peanut sauce	68.84	91.61	
		fried dishes, meat based	meat, prepared with spices, fried, steamed	beef	0	94.16	5.55
				cooking oil	95	5.84	
		fried dishes, poultry based	chicken, prepared with spices and flour, fried, steamed	chicken	0	68.49	16.97
				fried chicken seasoning	40.08	6.57	
				cooking oil	95	10.96	
				wheat	28.1	13.98	
			chicken, prepared with spices, fried, steamed	chicken	0	78.31	15.58
				fried chicken seasoning	40.08	9.16	
				cooking oil	95	12.53	
7	LEGUMES AND LEGUME PRODUCTS						
		composite food, oncom based	oncom based, stir fried	oncom	0	73.31	1.71
				stir-fry seasoning	5.36	26.42	
				basil	106	0.27	
		composite food, peanut based	peanut, with salted fish, fried	peanuts	220	54.46	131.76
				anchovy	3	34.04	
				cooking oil	95	11.5	
		composite food, tempeh based	tempeh in chili sauce	tempeh	161	43.15	87.05
				Balado spice	21.88	49.82	
				cooking oil	95	7.03	
			tempeh, fried	tempeh	161	80.65	148.23
				cooking oil	95	19.35	
			tempeh, in soy sauce soup, fried	tempeh	161	75.06	123.14
				seasoning sauce	9.21	24.94	
			tempeh, with soy sauce soup	tempeh	161	81.68	135.18
				flavoring stews	20.05	18.32	
			tempeh, with soy sauce, stir fried	red onion	5	16.09	24.1
				garlic	1	2.76	
				cayenne pepper	83	2.48	
				galangal	0	2.48	
				bay leaves	0	0	
				soy sauce	0	9.93	
				cooking oil	95	8.31	
				tempeh	7.1	57.95	
		composite food, tofu based	fried tofu, served with spice and sour sauce	tofu	#N/A	33.71	45.64
				peanut sauce	68.84	66.29	
			tofu in red pepper sauce	tofu	#N/A	42.73	14.47
				spicy seasoning	21.88	54.62	
				cooking oil	95	2.65	
			tofu, fried	tofu	#N/A	90.01	9.49
				cooking oil	95	9.99	
			tofu, in soy sauce soup, fried	tofu	#N/A	65.65	11.11
				seasoning sauce	9.21	25.09	
				cooking oil	95	9.26	
			tofu, steamed and spiced, wrapped in	tofu	#N/A	56.89	10.89

No	Food Group	Sub-Group	Food Item	Composition	PS Content (mg/100 g)	% composition in food item	Estimated PS content (mg/100 g food item)
			banana leaf	spiced seasoning	25.27	43.11	
			tofu, stir fried	tofu	#N/A	84.73	0.82
				stir-fry seasoning	5.36	15.27	
			tofu, with soy sauce soup	tofu	#N/A	78.65	4.28
				flavoring stews	20.05	21.35	
		legumes, mung bean based	mung bean porridge	green bean	23	23.58	15.41
				coconut milk	1	33.75	
				black rice	26.8	36	
				white bread	0	6.66	
		legumes, peanut based	peanut, fried	peanuts	220	88.42	205.52
				cooking oil	95	11.58	
8	SNACK FOODS						
		fried snacks, plantain based	chips, plantain, fried	banana	16	92.68	21.78
				cooking oil	95	7.32	
			plantain, wheat flour, fried	banana	16	75.02	23.69
				wheat	28.1	18	
				cooking oil	95	6.98	
		fried snacks, rice flour based	rice flour, fried	rice flour	0	92.76	6.87
				cooking oil	95	7.24	
			rice, fried	glutinous rice	35.5	60.24	59.16
				cooking oil	95	39.76	
			snacks, flour based, deep fried	composite flour	0	62.5	35.63
				cooking oil	95	37.5	
			traditional cake, rice flour with palm sugar, fried (kue cucur)	wheat	28.1	12.2	33.79
				corn flour	22.5	73.17	
				cooking oil	95	14.63	
		fried snacks, soy based	oncom based, with wheat flour, fried	oncom	0	60.31	100.23
				fried flour dough	9.29	56.27	
				cooking oil	95	100	
			tempeh based, wheat flour, fried	tempeh	161	68.62	133.53
				spices fried tempeh	17.44	8.72	
				cooking oil	95	22.66	
			tofu based, wheat flour, filled with sprout and vegetables, fried	tofu	#N/A	54.35	12.98
				fried flour dough	9.29	10.87	
				bean sprouts	15	17.39	
				carrots	12	8.7	
				cabbage	14.8	8.7	
				cooking oil	95	7.4	
			tofu based, wheat flour, fried	tofu	#N/A	75.97	11.54
				wheat	28.1	16.88	
				cooking oil	95	7.15	
		fried snacks, tuber based	cassava cake with oncom and spices, fried	cassava	#N/A	63.47	6.87
				oncom	0	29.29	
				cooking oil	95	7.24	
			cassava cake, fried	cassava	#N/A	93.46	6.21
				cooking oil	95	6.54	
			cassava, fried	cassava	#N/A	59	38.95
				cooking oil	95	41	
			chips, taro, fried	taro	19	97.85	20.64
				cooking oil	95	2.15	

No	Food Group	Sub-Group	Food Item	Composition	PS Content (mg/100 g)	% composition in food item	Estimated PS content (mg/100 g food item)
			sweet potato cake, fried	sweet potato	12	92.76	18.01
				cooking oil	95	7.24	
			sweet potato, fried	sweet potato	12	91.74	18.85
				cooking oil	95	8.26	
			taro, fried	taro	19	92.85	24.43
				cooking oil	95	7.15	
			traditional snack, cassava cake with palm sugar, fried	cassava	#N/A	77.3	6.87
				brown sugar	0	15.46	
				cooking oil	95	7.24	
		fried snacks, wheat flour based	bread, with chocolate or fruit jam filling, fried	white bread	0	80	10.7
				strawberry jam	12	10	
				cooking oil	95	10	
			doughnut, fried, served with margarine and chocolate rice topping	wheat	28.1	74.21	48.36
				margarine	146	11.13	
				meses	59	7.42	
				cooking oil	95	7.24	
			fried mix vegetable, with wheat flour, fried	cabbage	14.8	20.06	17.39
				carrots	12	20.06	
				leeks	19.4	2.51	
				fried flour dough	9.29	50.14	
				cooking oil	95	7.24	
			snacks, flour based, prepared with peanut, deep fried	peanuts	220	40.14	131.74
				wheat	28.1	20.07	
				cooking oil	95	39.8	
			snacks, flour based, prepared with salted fish, deep fried	wheat	28.1	47.85	18.97
				anchovy	3	47.85	
				cooking oil	95	4.31	
			traditional snack, wheat flour, fried	wheat	28.1	35.71	53.07
				margarine	146	17.86	
				cooking oil	95	17.86	
				eggs	0	10.71	
				granulated sugar	0	17.86	
			wheat flour based, banana and chocolate rice, fried	banana	16	45.71	31.64
				chocolate milk	59	19.05	
				wheat	28.1	30.48	
				cooking oil	95	4.76	
			wheat flour based, egg and vegetables, fried	wheat	28.1	10.64	37.02
				meat	0	35.46	
				leeks	19.4	1.77	
				cooking oil	95	35.46	
				eggs	0	16.67	
			wheat flour based, filled with rice noodle and vegetables, fried	wheat	28.1	45.71	10.09
				rice noodle	22.5	30.48	
				carrots	12	19.05	
				cooking oil	95	4.76	
			wheat flour based, filled with vegetables or egg, fried	wheat	28.1	48.59	24.28
				carrots	12	22.09	
				potato	5	22.09	
				cooking oil	95	7.24	
			wheat flour based, sweet corn, fried	wheat	28.1	56.22	33.09
				corn	28.5	36.54	
				cooking oil	95	7.24	
		other bakery wares	pancake, plain				
				wheat	28.1	5.39	1.58
				coconut milk	1	6.47	
			pancake, with cheese	wheat	28.1	55.56	42.65

No	Food Group	Sub-Group	Food Item	Composition	PS Content (mg/100 g)	% composition in food item	Estimated PS content (mg/100 g food item)
			and condensed milk	margarine	146	18.52	
				cheese	0	9.26	
				sugar	0	7.41	
				condensed milk	0	9.26	
			pancake, with peanut, chocolate rice, and sugar	wheat	28.1	60	73.96
				peanuts	220	10	
				chocolate milk	59	10	
				margarine	146	20	
			pancake, with sticky rice and condensed milk	wheat	28.1	54.55	66.75
				black rice	26.8	18.18	
				peanuts	220	9.09	
				margarine	146	18.18	
		traditional cake and snacks	traditional cake, cassava based, steamed (Ketimus)	cassava	#N/A	95.08	0
				brown sugar	0	4.92	
			traditional cake, pie, mung bean/chocolate/black bean filling (Kue Pia)	wheat	28.1	45.95	18.8
				corn flour	37	2.7	
				liquid milk	0	13.51	
				cooking oil	95	10.81	
				green bean	23	10.81	
				coconut milk	1	16.22	
			traditional cake, rice flour and grated coconut, pan fried (Kue Pancong)	shredded coconut	47	22.73	31.21
				corn flour	22.5	22.73	
				coconut milk	1	54.55	
			traditional cake, rice flour based, steamed (Kue Lopis)	glutinous rice	35.5	81.08	31.32
				brown sugar	0	13.51	
				coconut	47	5.41	
			traditional cake, rice flour, steamed (Kue Apem)	wheat	28.1	23.26	14.8
				corn flour	22.5	34.88	
				coconut milk	1	41.86	
			traditional cake, spekkoek, layer cake (Lapis Legit)	margarine	146	50	68.45
				wheat	28.1	15	
				milk powder	#N/A	5	
				sugar	#N/A	30	
			traditional cake, wheat flour and banana, steamed (Nagasari)	banana	16	50	8
				flour	0	50	
			traditional cake, wheat flour pan fried (Kue Ape)	wheat	28.1	22.22	13.55
				corn flour	22.5	11.11	
				coconut milk	1	66.67	
**9**	**VEGETABLES AND VEGETABLE PRODUCTS**						
		composite food, mix vegetables soup	mix all vegetables soup, soup only	red onion	5	73.19	6.61
				garlic	1	11.7	
				leeks	19.4	10.54	
				celery	6	3.51	
				pepper powder	55	1.05	
			mix all vegetables, served sour soup	yam beans	10	26.98	28.72
				peanuts	220	7.08	
				cucumber	14	24.77	
				cabbage	14.8	10.61	
				bean sprouts	15	28.75	
				pickled sauce	60.36	1.82	
			mix all vegetables, served with vinegar and onion	cucumber	14	31.25	19.75
				carrots	12	31.25	
				cayenne pepper	83	12.5	

No	Food Group	Sub-Group	Food Item	Composition	PS Content (mg/100 g)	% composition in food item	Estimated PS content (mg/100 g food item)
				red onion	5	25	
			mix all vegetables, soup	cabbage	14.8	16.39	12.83
				bean	35	13.66	
				leeks	19.4	1.71	
				potato	5	21.85	
				tomatoes	7	6.49	
				carrots	12	20.48	
				soup seasoning	6.61	19.43	
			mix vegetables in coconut milk soup	chickpea	35	11.78	44.83
				eggplant	7	9.82	
				sweet corn	28.5	21.28	
				peanuts	220	13.75	
				chayote	12	11.78	
				melinjo	#N/A	3.93	
				jackfruit	#N/A	9.82	
				leaves melinjo	#N/A	2.95	
				vegetable soup lodeh	15.44	14.9	
			mix vegetables soup, with chicken/meat/meatball added	cabbage	14.8	11.62	9.1
				bean	35	9.69	
				leeks	19.4	1.21	
				potato	5	15.5	
				tomatoes	7	4.6	
				carrots	12	14.53	
				soup seasoning	6.61	13.78	
				chicken	0	29.06	
			Mixed vegetables sour soup	chickpea	35	10.98	41.12
				leaves melinjo	#N/A	2.74	
				sweet corn	28.5	19.82	
				peanuts	220	12.8	
				chayote	12	10.98	
				melinjo	#N/A	3.66	
				jackfruit	#N/A	10.98	
				papaya	NA	14.18	
				sour soup	15.44	13.87	
			rice noodle and sprout spicy soup, with or without chicken/prawn/meat/egg added (laksa)	bean sprouts	15	27.27	10.68
				rice noodle	22.5	27.27	
				coconut milk	1	45.45	
		composite food, mix vegetables, steamed	mix all vegetables, steamed, served with grated coconut	papaya leaf	#N/A	41.67	7.83
				cassava leaves	#N/A	41.67	
				shredded coconut	47	16.67	
		composite food, mix vegetables, stir fried	mix all vegetables, stir fried	seasoning	5.96	68.74	10.83
				bean	35	8.33	
				corn	28.5	8.33	
				carrots	12	8.33	
				tomatoes	7	6.25	
		dishes with peanut sauce, mix vegetable	mix all vegetables, served with peanut sauce (Gado-gado)	tofu	#N/A	7.73	34.8
				Chickpea	35	11.86	
				cucumber	14	4.38	
				bean sprouts	15	9.54	
				chayote	12	15.46	
				carrots	12	3.61	
				kale	9	5.93	
				cabbage	14.8	5.15	
				peanut sauce	68.84	36.34	

No	Food Group	Sub-Group	Food Item	Composition	PS Content (mg/100 g)	% composition in food item	Estimated PS content (mg/100 g food item)
			mix all vegetables, served with peanut sauce (Karedok)	cabbage	14.8	15.29	41.25
				cucumber	14	14.37	
				green eggplant	7	4.59	
				basil	106	2.14	
				longbean	35	11.01	
				sweet potato	12	2.75	
				bean sprouts	15	7.65	
				peanut sauce	68.84	42.2	
			mix all vegetables, served with peanut sauce (Pecel)	carrots	12	23.08	61.04
				peanut sauce	200.11	27.47	
				chayote	12	27.47	
				papaya	NA	21.98	
		vegetables, processed, fried	potato in chili sauce, with or without chicken oval	potato	5	65.04	5.49
				spicy seasoning	6.39	34.96	
			potato, mashed, battered with egg, fried	potato	5	76.61	9.51
				eggs	0	17.41	
				cooking oil	95	5.98	
			sweet potato, mashed, fried	taro	19	68.43	28.22
				eggs	0	15.55	
				cooking oil	95	16.01	
		vegetables, processed, steamed or boiled	bamboo shoots, stir fried	bamboo shoot	19	54.55	10.82
				coconut milk	1	45.45	
			cassava leaves, with soup	cassava leaves	#N/A	55.88	0.44
				coconut milk	1	44.12	
			cassava, boiled, with soy sauce soup	potato	5	79.08	8.15
				flavoring stews	20.05	20.92	
			cassava, steamed, mashed, served with grated coconut	cassava	#N/A	92.86	3.36
				coconut	47	7.14	
			Chinese cabbage, boiled, with soup	Chinese cabbage	13	80.19	11.41
				soup seasoning	4.95	19.81	
			cucumbers, stir fried	cucumber	14	61.66	21.96
				soup seasoning	34.77	38.34	
			jackfruit, boiled, with coconut milk soup	jackfruit	#N/A	64.35	2.15
				soup seasoning	6.03	35.65	
			katuk leaf, soup	katuk	9	74.91	8.3
				soup seasoning	6.2	25.09	
			mushroom, soup	soup seasoning	3.85	28.13	2.52
				mushrooms	2	71.87	
			oyong, soup	oyong	24	46.09	18.16
				rice noodle	22.5	23.04	
				katuk sauce	6.2	30.87	
			spinach, boiled, with soup, with/without corn added	spinach	9	72.02	8.2
				spinach soup	6.15	27.98	
			sweet potato, boiled with palm sugar and coconut milk	sweet potato	12	49.26	6.42
				coconut milk	1	50.74	
			taro, steamed, mashed, served with grated coconut	taro	19	95.74	20.19
				coconut	47	4.26	
		vegetables, processed, stir fried	beans, broadbeans, green, stir fried	bean	35	46.44	39.6
				stir-fry seasoning	5.36	53.56	
			beans, longbean, stir	longbean	35	73.51	27.15

No	Food Group	Sub-Group	Food Item	Composition	PS Content (mg/100 g)	% composition in food item	Estimated PS content (mg/100 g food item)
			fried	stir-fry seasoning	5.36	26.49	
			bitter gourd, stir fried	bitter gourd	#N/A	67.55	1.74
				stir-fry seasoning	5.36	32.45	
			brassica leaves, stir fried	seasoning	4.95	17.19	23.35
				cooking oil	95	10	
				Brassica leaves	13	100	
			broccoli, stir fried	broccoli	36.7	60.95	24.46
				stir-fry seasoning	5.36	39.05	
			cabbage, stir fried	cabbage	14.8	62.48	11.26
				stir-fry seasoning	5.36	37.52	
			carrot, stir fried	carrots	12	79.24	10.62
				stir-fry seasoning	5.36	20.76	
			cauliflower, stir fried	cauliflower	18	58.12	12.71
				stir-fry seasoning	5.36	41.88	
			chayote, stir fried	stir-fry seasoning	5.36	21.54	13.67
				cooking oil	95	3.74	
				chayote	12	74.72	
			eggplant in chili sauce	eggplant	7	42.08	15.62
				spicy seasoning	21.88	57.92	
			eggplants, stir fried	eggplant	7	67.55	6.47
				stir-fry seasoning	5.36	32.45	
			fern leaves, stir fried	fern leaves	#N/A	66.41	1.8
				stir-fry seasoning	5.36	33.59	
			sprout, stir fried	bean sprouts	15	64.87	11.08
				soup seasoning	3.85	35.13	
			water spinach, stir fried	stir-fry seasoning	5.36	37.87	17.01
				cooking oil	95	10.91	
				kale	9	51.22	
			yellow velvetleaf, stir fried	velvetleaf	#N/A	59.87	2.15
				stir-fry seasoning	5.36	40.13	
